# Supplementary material for: Disease prediction with multi-omics and biomarkers empowers case–control genetic discoveries in the UK Biobank
Source: Nat Genet. 2024 Sep 11;56(9):1821–31. doi: 10.1038/s41588-024-01898-1 (PMC11390475; doi:10.1038/s41588-024-01898-1)
Supplement: Supplementary file 1 — Supplementary Notes, references and Figs. 1–30. [file 41588_2024_1898_MOESM1_ESM.pdf]

nature genetics

Supplementary information https://doi.org/10.1038/s41588-024-01898-1

Disease prediction with multi-omics and biomarkers empowers case–control genetic discoveries in the UK Biobank

In the format provided by the authors and unedited

Supplementary Notes

MILTON disease prediction performance across ancestries and time models

Owing to the predominance of samples deriving from individuals of European (EUR) ancestry, models trained on these samples produced results for the highest number of ICD10 (International classification of diseases 10th Revision[1]) codes (Supplementary Table 20) across all three time-models, while models trained on Admixed American ancestry samples produced results for only 5 ICD10 codes (time-agnostic model: E78, Z86, E780, I10, R10; Supplementary Table 1b). Similarly, time-agnostic and prognostic models yielded more ICD10 codes than diagnostic models since more individuals received diagnoses for the studied phenotypes after biomarker (sample) collection, as compared to before, in the UK Biobank[2] (UKB, Fig. 2a).

MILTON benchmark with polygenic risk scores

For the comparison with each of the 28 disease-specific polygenic risk scores (PRS)[3], we mapped the UKB disease terms that have an associated PRS, to the corresponding ICD10 codes using string matching followed by manual curation (Supplementary Table 17). We observed that time-agnostic models trained on 67 quantitative traits significantly outperformed those trained on a single disease-specific PRS + sex + age for 111 out of 151 ICD10 codes (median $AUC_{67\ traits}$ vs $AUC_{disease\text{-}specific\ PRS}$: 0.71 vs 0.66, Mann-Whitney U two-sided $p=2.71\times10^{-8}$; Table 1, Fig. 3c, Supplementary Fig. 3a, Supplementary Table 4a).

For prognostic models, we observed that MILTON trained on 67 quantitative traits significantly outperformed those trained on a single disease-specific PRS (including sex and age as covariates) for 86 out of 121 ICD10 codes using prognostic models (median $AUC_{67\ traits}$ vs $AUC_{disease\text{-}specific\ PRS}$: 0.71 vs 0.65, Mann-Whitney U two-sided $p=7.10\times10^{-7}$). We observed the same trend for 65 out of 82 ICD10 codes using diagnostic models (median $AUC_{67\ traits}$ vs $AUC_{disease\text{-}specific\ PRS}$: 0.75 vs 0.67, Mann-Whitney U two-sided $p=2.03\times10^{-6}$; Table 1, Supplementary Fig. 3a-b, Supplementary Table 4a). PRS, however, had higher predictive performance than the 67 traits for certain solid cancer types, Alzheimer's disease (G30) and multiple sclerosis (G35) in prognostic and time-agnostic models.

MILTON training and performance leveraging plasma proteomics data

Based on the given cohort size limitations with available proteomics data and requiring a minimum of 100 diagnosed cases per phenotype for training, we were able to run MILTON employing the UKB plasma proteomics data for 1,299, 864 and 435 phenotypes under time-agnostic, prognostic and diagnostic time-models, respectively (Supplementary Table 1c). In addition to the diseases mentioned in main text that benefited most from the inclusion of proteomics data, of note, the three ICD10 chapters pertaining to mental and behavioural disorders, diseases of the circulatory system and diseases of the digestive system also contained diseases which largely benefitted from the addition of proteomics data ($AUC_{3k\ proteins+67\ traits} - AUC_{67\ traits} > 0.02$; Supplementary Fig. 3d).

Feature importance learnt by MILTON across ancestries and inferred disease clusters

Across 149 ICD10-codes that achieved AUC > 0.6 in all three ancestries, we observed that European (EUR) and African (AFR) ancestries as well as EUR and South Asian (SAS) ancestries have (a median of) 4 top features in common, while AFR and SAS share 3 top features (Fig. 4a). Two examples with high

overlap of top features across ancestries are Type 1 diabetes mellitus (E10: $AUC_{\text{all three ancestries, time-agnostic model}}$ = 0.93 ± 0.04; top shared features: EUR-AFR=6, EUR-SAS=5, AFR-SAS=6) and Chronic renal failure (N18: $AUC_{\text{all three ancestries, time-agnostic model}}$ = 0.82 ± 0.03; top shared features: EUR-AFR=5, EUR-SAS=6, AFR-SAS=5).

Although for certain diseases presence of clinically used diagnostic biomarkers led to superior performance, we also noticed several high performing phenotypes (AUC>0.7) where the top biomarkers are not typically clinically used for diagnosis. An example is schizophrenia that lacks a diagnostic test clinically, but MILTON trained on 67 traits achieved an $AUC_{\text{prognostic, 67 traits}}$ of 0.8 (vs $AUC_{\text{prognostic, PRS}}$ = 0.65) with pulse rate[4–6] and forced expiratory volume in 1-second (FEV1)[7] as top predictive features. Another example is bipolar affective disorder where blood tests are currently not used for diagnosis, but models trained on 67 traits achieved an $AUC_{\text{prognostic, 67 traits}}$ of 0.74 compared to models trained on corresponding PRS ($AUC_{\text{prognostic, PRS}}$=0.65). In such cases, where top predictive biomarkers currently lack known biological connection with a disease, we suggest these links to be explored further as our knowledge of disease-specific biomarkers keeps increasing.

Furthermore, we generated groups of phenotypes enriched for similar biomarker profiles, using Louvain clustering, and visualized them on a 2-dimensional t-SNE plot (Fig. 4e, Supplementary Table 6a). Focusing on one of the clusters that contains E10: Type 1 dependent diabetes mellitus (Fig. 4d) as an example, we checked whether individuals diagnosed with this ICD10 code are also diagnosed with other ICD10 codes in the same cluster (see Methods; Supplementary Table 6b). We observed that 22 out of 35 codes in this cluster were already significantly enriched ($p<=5.1x10^{-48}$) as comorbidities in patients diagnosed with E10 (Supplementary Fig. 30c, Supplementary Table 6b).

Enhanced PheWAS analysis with augmented MILTON cohorts

For this analysis, instead of looking at each individual rare variant, we combine rare variants in each gene with similar effects together and compare the aggregated presence of these rare-variants in cases with controls[8]. To minimise redundancies, we mapped all associations with ICD10 sub-codes such as N18.0, N18.1 to their 3-character parent code (N18 in this case). This resulted in 1,465 significant gene-ICD10 associations ($p_{MILTON}<1x10^{-8}$) between 451 3-character ICD10 codes and 165 genes across non-synonymous qualifying variants (QVs)[9] in EUR ancestry. We also tested employing MILTON predicted probability scores as continuous measures instead of using dichotomised cohorts for subsequent rare-variant collapsing analyses. However, sensitivity and specificity were low (see Methods), and thus, we retained the dichotomised augmented cohorts as the default approach.

We have previously demonstrated that careful cohort harmonisation, as applied here, minimises population stratification in the UKB and controls genomic inflation[9]. To verify this holds true for the four Milton-generated cohorts, we calculated the genomic inflation factor for each QV model for five diseases with varying prevalence across Europe. The null distributions were calculated with n-1 case-control permutation. Across the 275 genomic inflation factors calculated (11 QV models, 5 diseases, 5 cohorts), the $lambda_{mean}$ = 1.07±0.05 (Supplementary Table 18).

With regards to the characterisation of 'known binary' associations from the enhanced PheWAS analysis, any gene-disease associations from ICD10-codes where MILTON had no contribution to case cohort extension were excluded from the reported baseline associations. The 'known binary' associations constituted a smaller percentage of total associations identified in each cohort as we moved from L0 to L3 cohorts, consistent with our expectations that the case-prediction signals may

diminish as a function of relaxing the MILTON probability inclusion threshold (Supplementary Fig. 7c-d).

We then investigated the effect of highly predictive biomarker features per ICD10-code on the remaining 1,229 gene-ICD10 associations, to detect any putative novel signals that may be reflecting correlation with a biomarker rather than independent disease association. To this end, we take into consideration any genes achieving genome-wide significance with a biomarker in the baseline quantitative PheWAS and examine whether these biomarkers are among the top predictors for a given phenotype in the MILTON cohorts. To identify these overlaps, we first shortlisted features per ICD10-code that had feature importance z-score (FIZ) greater than 1.2 (see Methods; Supplementary Fig. 7e-f). We then labelled all genes associated with a given ICD10-code that were also significantly associated with its highly predictive feature(s) in quantitative PheWAS results from Wang et al.[9] ($p_{quant\ PheWAS\ for\ FIZ>1.2}<1x10^{-8}$) as "known quant". These accounted for 1,047 significant gene-disease associations which we do not consider as novel ($p_{MILTON}<1x10^{-8}$ & $p_{Baseline}>1x10^{-8}$ & $p_{quant\ PheWAS\ for\ FIZ>1.2}<1x10^{-8}$; Table 2). Finally, 182 putative novel associations were reported by MILTON, which refer to 3-character level ICD10 codes, to reduce over-reporting of novel associations across similar diseases.

As an additional validation, we evaluated MILTON's performance on several known gene-disease associations, as a positive control. For example, the association of protein truncating variants (PTVs) in *SCN5A (*sodium voltage-gated channel alpha subunit 5 gene*)* with Atrioventricular and left bundle-branch block (I44)[10] achieved a p-value of 7.78×10^{-8} in baseline cohort but a p-value of $4.10x10^{-9}$ in MILTON L0 cohort under the time-agnostic model. Compared to baseline, the number of cases with PTVs increased from 26 to 40 in the L0 cohort and the number of sex-matched controls with PTVs only increased from 173 to 174. Similarly, the association of PTVs in multiple endocrine neoplasia type 1 (*MEN1*) gene with D35 benign neoplasm of other and unspecified endocrine glands[11,12] had a p-value of 2.88×10^{-5} in baseline cohort but a p-value of $2.46x10^{-11}$ in L3 cohort under time-agnostic model. The total number of D35 cases used for rare-variant collapsing analysis increased from 1,964 to 13,986 in L3 cohort with an increase in cases with PTVs in *MEN1* from 3 (0.14% carrier frequency) to 9 (0.07% carrier frequency) and decrease in controls with PTVs in *MEN1* from 9 (0.0022%) to 4 (0.0010%).

Prioritising individuals with lower polygenic risk scores

There is evidence that individuals with a given disease, despite being characterised by a lower disease-specific PRS, they may still have higher rare-variant burden[13]. We therefore checked if adjusting our predicted probability scores with PRS may help to prioritize these individuals, thus improving yield. To do this, we repeated our rare-variant collapsing analysis on L0 cohorts derived from probability scores divided by 1+PRS for 35 ICD10-codes that had disease-specific PRS available. We observed a mixed trend (Supplementary Fig. 11a, Supplementary Table 19), which showed that out of 16 baseline gene-ICD10 associations related to protein truncating variants ($p<10^{-8}$), 1 association was missed out in both cohorts ($p>10^{-8}$), 7 were boosted in adjusted cohorts (Supplementary Fig. 11b) while 8 were boosted in unadjusted cohorts (Supplementary Fig. 11c). Therefore, certain diseases such as Type 2 diabetes mellitus (E11) may benefit from this approach while others may not. Apart from those recovered from baseline cohorts, PheWAS on PRS-adjusted and unadjusted MILTON cohorts also resulted in 27 and 28 genome-wide significant associations ($p<1x10^{-8}$), respectively, with an overlap of 22 associations (Supplementary Table 19).

PheWAS collapsing analysis on prediction scores as continuous traits

To perform collapsing analysis, we used the procedure described in Quanli et al. 2021[9]. Specifically, we aggregated variants within each gene that fitted a given set of criteria, identified as QVs. In total, we performed nine non-synonymous collapsing analyses, including eight dominant and one recessive model, plus a tenth synonymous variant model that serves as an empirical negative control. In each model, for each gene, the proportion of cases was compared with the proportion of controls for individuals carrying one or more QVs in that gene. The exception is the recessive model, where a participant must have two qualifying alleles, either in homozygous or potential compound heterozygous form. Hemizygous genotypes for the X chromosome were also qualified for the recessive model. These models vary in terms of allele frequency (from private up to a maximum of 1%), predicted consequence (for example, PTV or missense) and REVEL and MTR scores. Based on SnpEff annotations, we defined synonymous variants as those annotated as 'synonymous_variant'. We defined PTVs as variants annotated as exon_loss_variant, frameshift_variant, start_lost, stop_gained, stop_lost, splice_acceptor_variant, splice_donor_variant, gene_fusion, bidirectional_gene_fusion, rare_amino_acid_variant and transcript_ablation. We defined missense as: missense_variant_splice_region_variant and missense_variant. Non-synonymous variants included: exon_loss_variant, frameshift_variant, start_lost, stop_gained, stop_lost, splice_acceptor_variant, splice_donor_variant, gene_fusion, bidirectional_gene_fusion, rare_amino_acid_variant, transcript_ablation, conservative_inframe_deletion, conservative_inframe_insertion, disruptive_inframe_insertion, disruptive_inframe_deletion, missense_variant_splice_region_variant, missense_variant and protein_altering_variant.

For all models, we applied the following quality control filters: minimum coverage 10×; annotation in consensus coding sequence (CCDS) transcripts (release 22; approximately 34 Mb); at most 80% alternate reads in homozygous genotypes; percentage of alternate reads in heterozygous variants greater than or equal to 0.25 and less than or equal to 0.8; binomial test of alternate allele proportion departure from 50% in heterozygous state $P > 1 \times 10{-}6$; $GQ \geq 20$; $FS \leq 200$ (indels), $FS \leq 60$ (SNVs); $MQ \geq 40$; $QUAL \geq 30$; $RPRS \geq -2$; $MQRS \geq -8$; DRAGEN variant status = PASS; the variant site achieved tenfold coverage in ≥25% of gnomAD exomes; and, if the variant was observed in gnomAD exomes, the variant achieved exome z-score ≥ -2.0 and exome $MQ \geq 30$.

We have created a phenotype dataset based on the MILTON scores, which serves as a quantitative trait, and conducted association tests. Gene-based P-values were calculated using linear regression, while accounting for age and sex as covariates.

Association tests were performed using a custom framework, PEACOK (PEACOK 2.0.0), which is an extension and enhancement of PHESANT. PEACOK 2.0.0 is available on GitHub: https://github.com/astrazeneca-cgr-publications/PEACOK/.

Genome-wide association studies (GWAS) on MILTON augmented cohorts

In our GWAS results on 20 random phenotypes, stratified based on MILTON's AUC across the phenome (Supplementary Table 9b), we first checked whether there was concordance on the sign of Z-scores between the baseline and the MILTON cohorts and if Z-scores were preferentially boosted in the MILTON cohorts. Indeed, we observed that ~96% of all Z-scores were directionally concordant across the L0-L3 cohorts, and 84.1%-89.7% (respectively) among them had a boosted Z-score value (Supplementary Fig. 14a). Furthermore, the majority of all GWAS signals (84.7%-90.1% across L0-L3) were boosted in MILTON cohorts compared to baseline (Supplementary Fig. 14b). Importantly, most of the baseline genome-wide significant signals were also significant in the L0-L2 cohorts (88.1%,

85.1% and 82.2% respectively; Supplementary Fig. 14c). As expected, the L3 cohort had a slightly lower overlap (69.3%). Among the significant putative novel hits identified in the L0 and L1 cohorts, most (88.9% and 59.6%, respectively) were also nominally significant in the baseline cohort (p<0.01; Supplementary Fig. 14d). The L2-L3 cohorts had slightly lower support (44.6% and 28.6%, respectively). These results demonstrate that the MILTON cohorts overall boost the significance of common variant signals. Because 26% of the GWAS hits arose from a single phenotype (E11, Type 2 diabetes mellitus), we repeated the validation analysis excluding this phenotype. The concordance/boosting of Z-scores and p-value significance were slightly higher than when including all phenotypes (Supplementary Fig. 15), thus demonstrating the validity of our findings across the range of examined phenotypes.

Finally, we compared the improvement in Z-score gains from MILTON (L0-L3) to those that would have been theoretically expected in the setting where we had increased sample sizes and perfect case/control classifications (see Supplementary Note section "Computing expected Z-scores for Milton Cohorts"). To achieve this, we fit two regression lines: one between "known Z" and "observed Z", and one between "known Z" and "theoretical Z". When averaging the signals across the 20 ICD10 codes, the observed fitted line shows an increase in gains compared to the equivalence line (y=x) but remains below the expected maximum defined by the theoretical fitted line (Supplementary Fig. 16). We repeated the same analysis just for the E11 ICD10 code (Type 2 Diabetes Mellitus) and observed the MILTON L0 and L1 cohorts achieve gains equivalent or comparable to the theoretically expected (Supplementary Fig. 17). Interestingly, when applying this analysis to certain ICD10 codes such as E16 Other disorders of pancreatic internal secretion (Supplementary Fig. 18), the observed gains match the theoretical ones on the L0-L2 cohorts and almost match the gains of the L3 cohort too. These results indicate that, overall, we achieve a good enrichment of true cases in the MILTON cohorts, with a potential dilution of signal in the L3 cohort for certain phenotypes.

Computing expected Z-scores for Milton Cohorts

Consider a set of GWAS summary statistics from the baseline `known' cohort for a binary trait for a variant *s*. Here $\hat{\beta}$ is an estimate of the genotype coefficient of *s* under the additive model and $\hat{\sigma}$ is the standard error associated with $\hat{\beta}$. It is then standard to compute a *Z* score summarising the association as:

$$Z = \frac{\hat{\beta}}{\hat{\sigma}}$$

Under this framework $\hat{\sigma}$ can be thought to consist of two components one relating to the minor allele frequency of *s*, $\widehat{\sigma_f}$ and another $\widehat{\sigma_N}$ that relates to sample size, such that:

$$Z = \frac{\hat{\beta}}{\widehat{\sigma_f}\widehat{\sigma_N}}$$

If we assume that β and σ remains constant for *s* from our baseline `known' cohort, an estimate of the expected Z scores as we alter case/control size configurations using Milton can be inferred by first computing $\widehat{\sigma_f}$ in the `known' cohort for *s* using:

$$\widehat{\sigma_f} = \frac{\hat{\beta}}{Z\widehat{\sigma_N}} = \sqrt{\left[2\hat{f}(1-\hat{f})\right]^{-1}}$$

where $\hat{\hat{f}}$ is the estimated MAF of *s* allowing for $\widehat{\sigma_f}$ to be estimated where $\hat{f}$ is unknown. Furthermore $\widehat{\sigma_N}$ for a case/control study can be estimated as:

$$\widehat{\sigma_N} = \sqrt{\frac{N_0 + N_1}{N_0 N_1}}$$

where N_0 and N_1 are the number of controls and cases respectively. This means that given a new case/control configuration under the strong assumptions of perfect classification between cases and controls and fixed estimates for $\hat{\beta}$ and $\widehat{\sigma_f}$ we can derive an expression to estimate Z', the *Z* score for *s* under this new configuration, as:

$$Z' = \frac{\hat{\beta}}{\widehat{\sigma_f}} \sqrt{\frac{N_0' N_1'}{N_0' + N_1'}}$$

Where N_0' and N_1' are the number of controls and cases for the new case/ control configuration respectively.

Validation with FinnGen Biobank

We aimed to identify support for the MILTON putative novel hits in external datasets, and specifically in the FinnGen Biobank[14], which has variant-level enrichment results available. Among imputed genotypes in FinnGen, 37% (n=3,656) of the MILTON putative novel ExWAS associations can be tested in FinnGen. Of these 3,656 associations, 54.76% (n=2,002) achieved p<0.05 in FinnGen[14] release 10 (Fig. 6d; Supplementary Table 8a and Supplementary Table 9a). For reference, among the genome-wide significant hits inferred in baseline PheWAS, 63.62% (n=5,098) can be tested in FinnGen of which 88.90% (n=4,532) had supporting evidence (p<0.05, Fig. 6d).

For validation of common variant-to-ICD10 associations obtained from GWAS, we investigated 14 ICD10-codes that can be mapped to summary statistics results in FinnGen Biobank[14] release 11 (Supplementary Table 8). After accounting for missing genotypes in FinnGen, 86.14% (n=87) of unique baseline associations ($p<1x10^{-8}$) could be tested in FinnGen of which 93.10% (n=81) had p<0.05 in FinnGen with same direction of effect (Fig. 6d, Supplementary Table 9b). Also, for 85.86% (n=77) of "known binary" associations ($p<1x10^{-8}$) that can be tested in FinnGen, 94.81% (n=73) associations had supporting evidence (p<0.05 with same direction of effect). Furthermore, 181 associations had $p<1x10^{-8}$ in extended cohorts but $p>1x10^{-8}$ in baseline cohorts. Of these, 14 associations were labelled as "known quantitative" based on previously known associations between closest gene to a given variant and most predictive feature(s) for a given ICD10-code. The remaining 167 associations were labelled as "putative novel" of which 38.92% (n=65) achieved p<0.05 in FinnGen with same direction of effect (Fig. 6d, Supplementary Table 9b).

Notably, we also observed that both baseline and putative novel gene-ICD10 associations had a higher number of significantly associated rare-variants (minor allele frequency < 0.01 with the given gene listed as one of its "nearest genes") with a given FinnGen phenotype (p<0.05) compared to randomly selected genes of same length, sampled 10 times (Supplementary Fig. 19; Supplementary Table 8b-c).

Putative novel hits rank highly in Mantis-ML 2.0

We sought to validate the putative novel hits inferred by PheWAS on MILTON augmented cohorts, using an independent machine learning tool, Mantis-ML 2.0[15,16], which is trained phenome-wide using graph neural networks on knowledge graphs. Mantis-ML 2.0 considers orthogonal, publicly available gene-centric resources such as the Human Phenotype Ontology (HPO), Open Targets and Genomics England as well as knowledge graphs to assign ranks for each gene in the human exome across thousands of phenotypes. Stepwise-hypergeometric tests were performed comparing Mantis-ML 2.0[16] predictions and associations identified by MILTON (Fig. 6a, see Methods). These tests aim not only to quantify the overlap between the two methods, which can be quantified by a simple Fisher's exact test, but also to rank the results in the assessment of the overlap enrichment. We performed these tests for fourteen phenotypes in Mantis-ML 2.0 where a mapping between human phenotype ontology[17] (HPO) and ICD10 was manually identified (Supplementary Table 16).

Notably, genes identified in L3 cohorts for non-synonymous QV models ($p<0.05$) were significantly enriched in top-ranking genes from Mantis-ML 2.0 compared to baseline cohort across all three time-models (Fig. 6a; comparison per phenotype is shown in Supplementary Fig. 20a). L3 is the largest of all augmented cohorts and contributed the highest number of putative novel associations across all four cohorts (Supplementary Fig. 7a,c,d).

Furthermore, all phenotypes were enriched in top-ranking genes under the ultra-rare damaging QV model (UR; UKB minor allele frequency ≤0.005%[9]) in MILTON augmented cohorts compared to synonymous QV model, followed by UR variants according to the missense tolerance ratio model (URmtr[9]) and protein truncating variant model (ptv[9]; Supplementary Fig. 20b-c). These validations pinpoint MILTON's power to highlight putative novel signals also supported by comprehensive AI-based gene-prioritisation methods, that leverage large amounts of biological evidence from literature and hundreds of public data resources.

Putative novel hits are enriched in AMELIE

As an additional assessment, we performed an automated literature search using AMELIE[18,19] (version 3.1.0), integrating nightly updates from the entire PubMED corpus, to estimate gene rankings for disease causality among a candidate gene pool. As part of the evaluation process, AMELIE requires diseases to be organised within the Human Phenotype Ontology (HPO). As such, it is necessary to exploit natural language processing (NLP) techniques to fully automate the procedure across a diverse range of disease-gene associations. More specifically, we found the 5 most semantically similar HPO diseases out of a pool of 17,451 phenotypes to each ICD10 code (at the 3- character level) and queried AMELIE for disease-gene associations for those diseases. We calculated semantic similarity through taking the Euclidean distance between the average BioWordVec[20] embeddings per disease term in the ICD10 and HPO disease terms.

The results of AMELIE for a given HPO-gene pair is a score (out of 100) representing the gene ranking. This is returned for up to 5 PubMed articles per disease-gene pair. We therefore aggregated (taking the mean) over these 5 articles to provide a single summary statistic of the rank of a disease-gene pair – taking into account that there are 5 HPO diseases per ICD10 code (found using NLP). We observed that for 13 out of 16 ICD10 chapters, putative novel targets (Supplementary Table 7a) had significantly higher AMELIE score ($p<0.05$) than negative controls generated through 10 samplings of random gene sets of same length (Fig. 6b). The top 10 phenotypes per chapter by AMELIE enrichment scores are shown in Supplementary Fig. 21.

Opportunities for future directions of MILTON

As new biobank initiatives continue to emerge and grow, we anticipate that longitudinal data will become available, enabling the use of additional methods, including autoencoders. UK Biobank is comprised of healthier, older individuals compared to UK population at large and its unique design ensures most biomarkers are measured for all individuals, minimizing missingness, which is eventually addressed via imputation by median (Supplementary Fig. 22). However, disparities may exist in biomarker capture across biobanks, where presence or absence of a biomarker may be indicative of the presence or absence of the diagnosis itself, inducing circularity in the predictions. Before applying any imputation in such cases, it would be important to first assess the missingness of data across all individuals and how it is stratified between cases and controls for each biomarker. The degree of missingness that makes a biomarker non-informative is biomarker and phenotype dependent, so it should be assessed on a case-by-case basis, considering the design of the Biobank and the phenotypes that are being examined. Finally, we have currently used the ICD10 hierarchy as our reference phenotype dataset, however, MILTON can be directly applied to any phenotyping configuration as can be the case with other biobanks.

References

1. International Classification of Diseases (ICD). https://www.who.int/standards/classifications/classification-of-diseases.
2. Bycroft, C. *et al.* The UK Biobank resource with deep phenotyping and genomic data. *Nature* **562**, 203–209 (2018).
3. Thompson, D. J. *et al.* UK Biobank release and systematic evaluation of optimised polygenic risk scores for 53 diseases and quantitative traits. 2022.06.16.22276246 Preprint at https://doi.org/10.1101/2022.06.16.22276246 (2022).
4. Quintana, D. S. *et al.* Reduced heart rate variability in schizophrenia and bipolar disorder compared to healthy controls. *Acta Psychiatr. Scand.* **133**, 44–52 (2016).
5. Benjamin, B. R. *et al.* Heart rate variability is associated with disease severity in psychosis spectrum disorders. *Prog. Neuropsychopharmacol. Biol. Psychiatry* **111**, 110108 (2021).
6. Resting vagal activity in schizophrenia: Meta-analysis of heart rate variability as a potential endophenotype | The British Journal of Psychiatry | Cambridge Core. https://www.cambridge.org/core/journals/the-british-journal-of-psychiatry/article/resting-

vagal-activity-in-schizophrenia-metaanalysis-of-heart-rate-variability-as-a-potential-endophenotype/7A76D9F7D23BD889E7CA39A98CE42A66.

7. Vancampfort, D. *et al.* Associations between expiratory spirometry parameters and limitations in daily life activities in patients with schizophrenia. *Gen. Hosp. Psychiatry* **36**, 172–176 (2014).
8. Povysil, G. *et al.* Rare-variant collapsing analyses for complex traits: guidelines and applications. *Nat. Rev. Genet.* **20**, 747–759 (2019).
9. Wang, Q. *et al.* Rare variant contribution to human disease in 281,104 UK Biobank exomes. *Nature* **597**, 527–532 (2021).
10. Schott, J.-J. *et al.* Cardiac conduction defects associate with mutations in SCN5A. *Nat. Genet.* **23**, 20–21 (1999).
11. Thakker, R. V. Multiple endocrine neoplasia type 1 (MEN1). *Best Pract. Res. Clin. Endocrinol. Metab.* **24**, 355–370 (2010).
12. Giraud, S. *et al.* Germ-Line Mutation Analysis in Patients with Multiple Endocrine Neoplasia Type 1 and Related Disorders. *Am. J. Hum. Genet.* **63**, 455–467 (1998).
13. Zhou, D. *et al.* Contextualizing genetic risk score for disease screening and rare variant discovery. *Nat. Commun.* **12**, 4418 (2021).
14. Kurki, M. I. *et al.* FinnGen provides genetic insights from a well-phenotyped isolated population. *Nature* **613**, 508–518 (2023).
15. Vitsios, D. & Petrovski, S. Mantis-ml: Disease-Agnostic Gene Prioritization from High-Throughput Genomic Screens by Stochastic Semi-supervised Learning. *Am. J. Hum. Genet.* **106**, 659–678 (2020).
16. Middleton, L. *et al.* Phenome-wide identification of therapeutic genetic targets, leveraging knowledge graphs, graph neural networks, and UK Biobank data. *Sci. Adv.* **10**, eadj1424 (2024).
17. Köhler, S. *et al.* The Human Phenotype Ontology in 2021. *Nucleic Acids Res.* **49**, D1207–D1217 (2021).

18. Birgmeier, J. *et al.* AMELIE speeds Mendelian diagnosis by matching patient phenotype and genotype to primary literature. *Sci. Transl. Med.* **12**, eaau9113 (2020).

19. Birgmeier, J. *et al.* AMELIE 3: Fully Automated Mendelian Patient Reanalysis at Under 1 Alert per Patient per Year. 2020.12.29.20248974 Preprint at https://doi.org/10.1101/2020.12.29.20248974 (2021).

20. Zhang, Y., Chen, Q., Yang, Z., Lin, H. & Lu, Z. BioWordVec, improving biomedical word embeddings with subword information and MeSH. *Sci. Data* **6**, 52 (2019).

Supplementary Figures

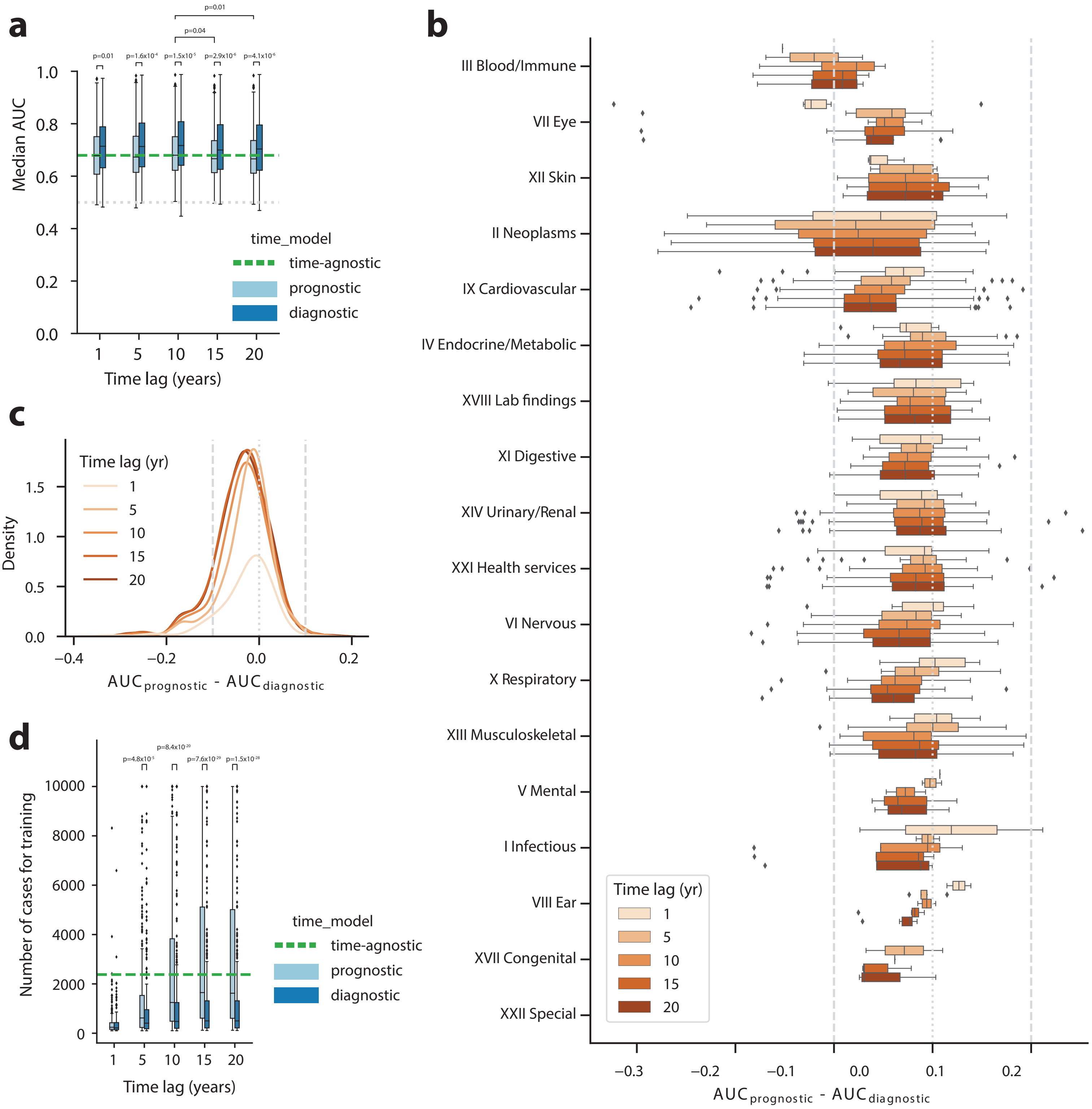

Supplementary Fig. 1 a Boxplots comparing the overall performance of MILTON-models across 400 random ICD10 codes when trained on cases satisfying the given time-lag and time-model. Gray, dashed line represent an AUC of 0.5, equivalent to random guesses. Number codes with results for (diagnostic, prognostic) time-models, respectively: 1 year (157, 192); 5 years (323, 371); 10 years (368, 371); 15 years (400, 400); 20 years (400, 399). Time-agnostic model had results for 400 codes. **b** Boxplots comparing the performance of MILTON-models across each ICD10-chapter when trained on cases satisfying the given time-lag and time-model for codes shown in panel **a** (1 year = 157 codes, 5 years = 323 codes, 10 years = 368 codes, 15 or 20 years = 400 codes). **c** Density plot showing the distribution of difference between prognostic and diagnostic models across ICD10 codes. **d** Boxplots comparing the number of cases satisfying the given time-lag available for training across 400 ICD10 codes. Mann-Whitney U test, two-sided p-values are shown in panels **a** and **d**. In panels **a**, **b** and **d**, the box-plot shows median as centre line, 25th percentile as lower box limit, 75th percentile as upper box-limit, whiskers extend to 25th percentile – 1.5 * interquartile range at the bottom and 75th percentile + 1.5*interquartile range at the top, points denote outliers.

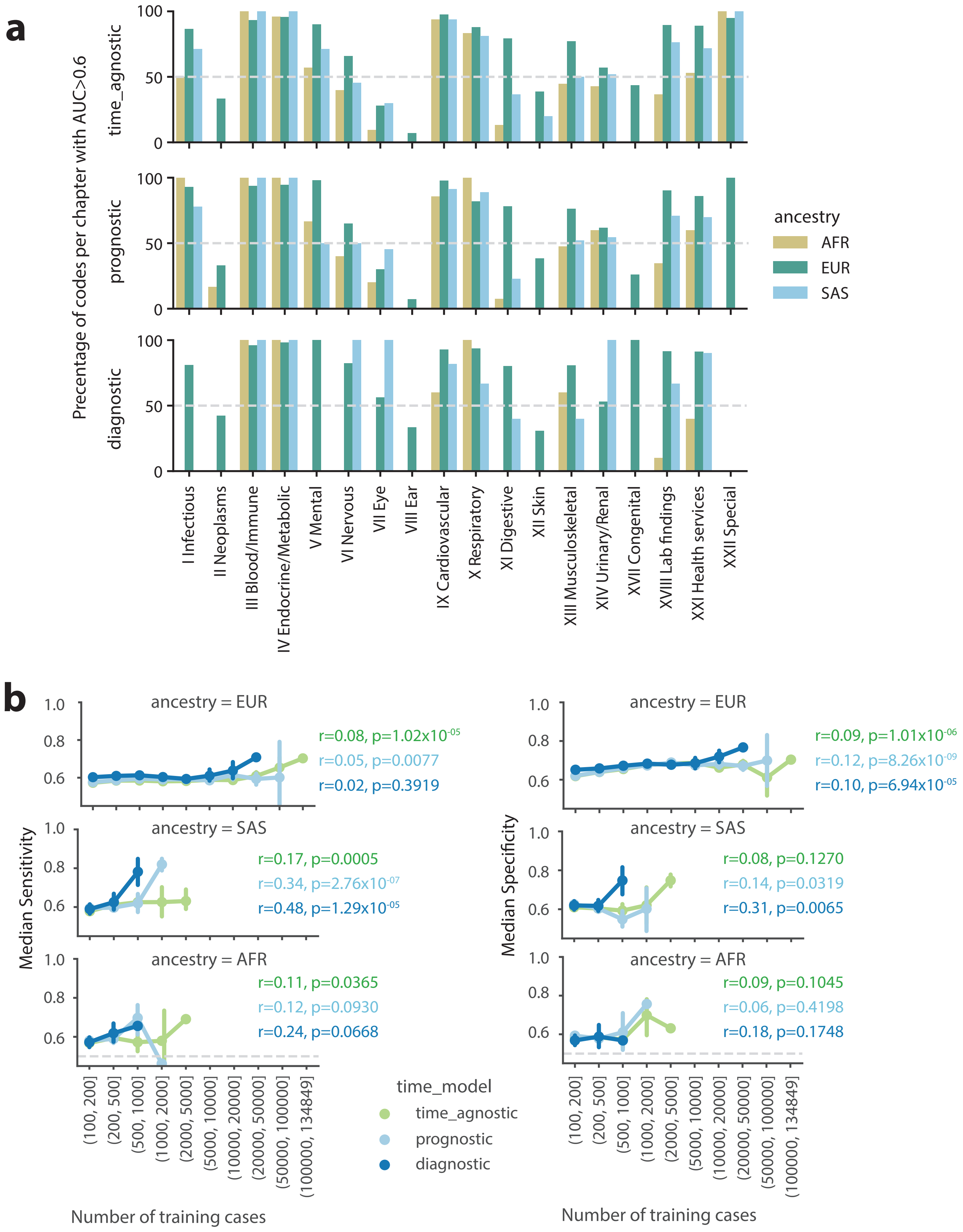

Supplementary Fig. 2 a Bar plots comparing the overall performance of MILTON-models across ICD10 chapters. Gray dashed line represents that 50% of ICD10 codes per chapter had AUC > 0.6. **b** Point-plots showing the variation of sensitivity and specificity with increasing number of cases available for training in each ICD10-code. The number of ICD10 codes in each ancestry are European (EUR) ancestry: $n_{time\_agnostic}$=3,196, $n_{prognostic}$=2,419 and $n_{diagnostic}$=1,547; African (AFR) ancestry: $n_{time\_agnostic}$=365, $n_{prognostic}$=194 and $n_{diagnostic}$=57; South Asian (SAS) ancestry: $n_{time\_agnostic}$= 412, $n_{prognostic}$=221 and $n_{diagnostic}$=76. Error-bar represents 95% confidence interval with centre representing mean statistic. Pearson correlation coefficients (r) and two-sided p-values (p) for each time-model are indicated.

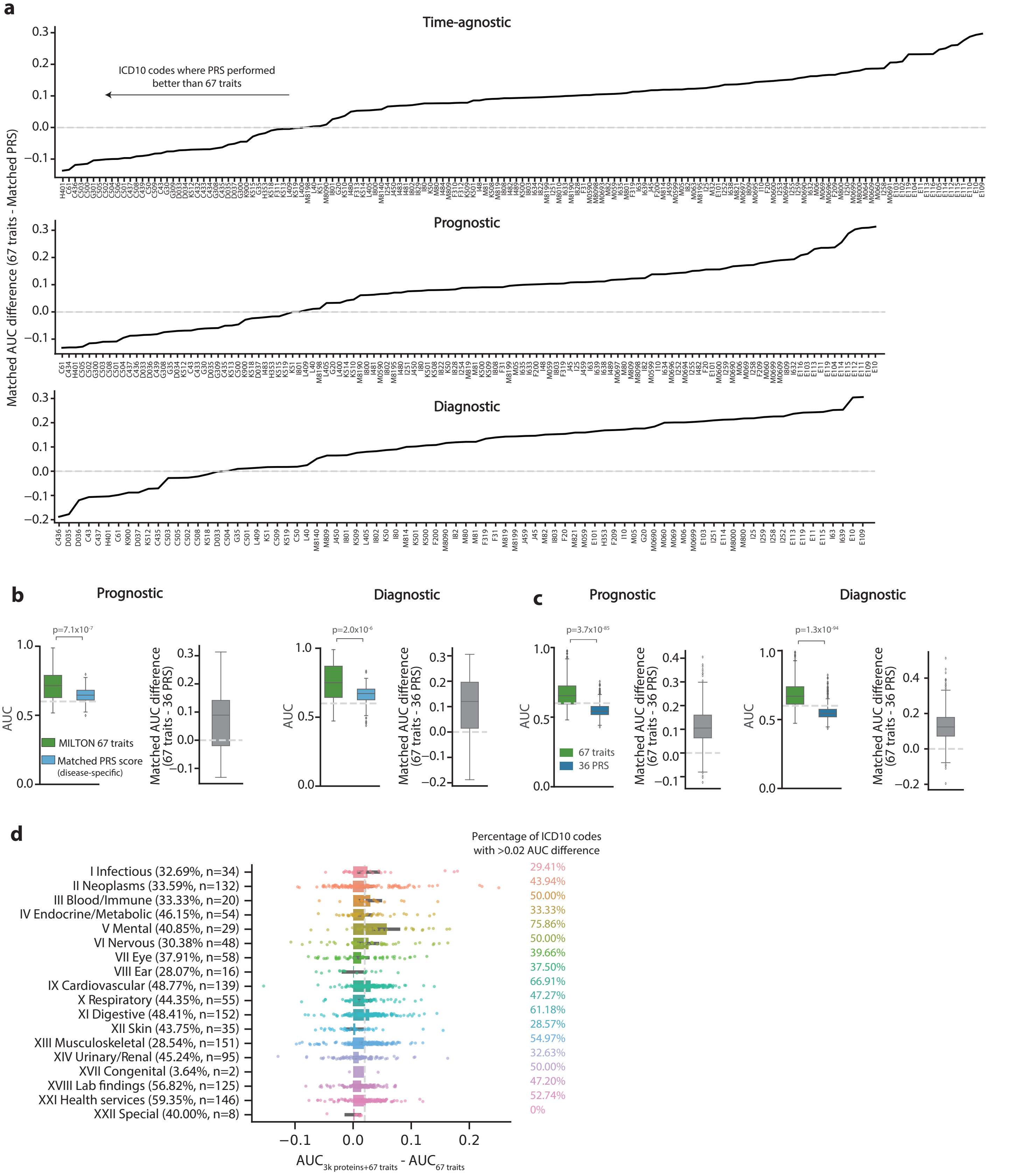

Supplementary Fig. 3 a Line-plots comparing the median AUC (y-axis) of MILTON models trained on 67 traits vs disease-specific PRS for each disease (x-axis). **b** Boxplots comparing the performance of MILTON prognostic models (left; n=121 ICD10 codes) and diagnostic models (right; n=82 ICD10 codes) trained on 67 traits vs disease-specific PRS. **c** Boxplots comparing the performance of MILTON prognostic models (left; n=499 ICD10 codes) and diagnostic models (right; n=500 ICD10 codes) trained on 67 traits vs 36 PRS. In panels **b** and **c**, Mann-Whitney U test two-sided p-values are shown and each box-plot shows median as centre line, 25th percentile as lower box limit, 75th percentile as upper box-limit, whiskers extend to 25th percentile – 1.5 *interquartile range at the bottom and 75th percentile + 1.5*interquartile range at the top, points denote outliers. **d** Bar plots showing the aggregated difference in MILTON's performance across chapters when models are trained on UKB plasma proteins + 67 traits vs 67 traits only; time-agnostic model and EUR ancestry. Please note that UKB plasma proteomics data is only available for a subset of individuals and MILTON was trained on these individuals only. The percentage (and number 'n') of ICD10 codes per chapter that had results in this reduced dataset compared to original dataset is given in the y-label. The percentage of ICD10 codes per chapter that had results in this reduced dataset and had an increase in AUC by at least 0.02 units upon addition of plasma protein abundance data is given on the right. Each bar denotes mean statistic with error bars denoting 95% confidence interval. Each color denotes the corresponding chapter on y-axis.

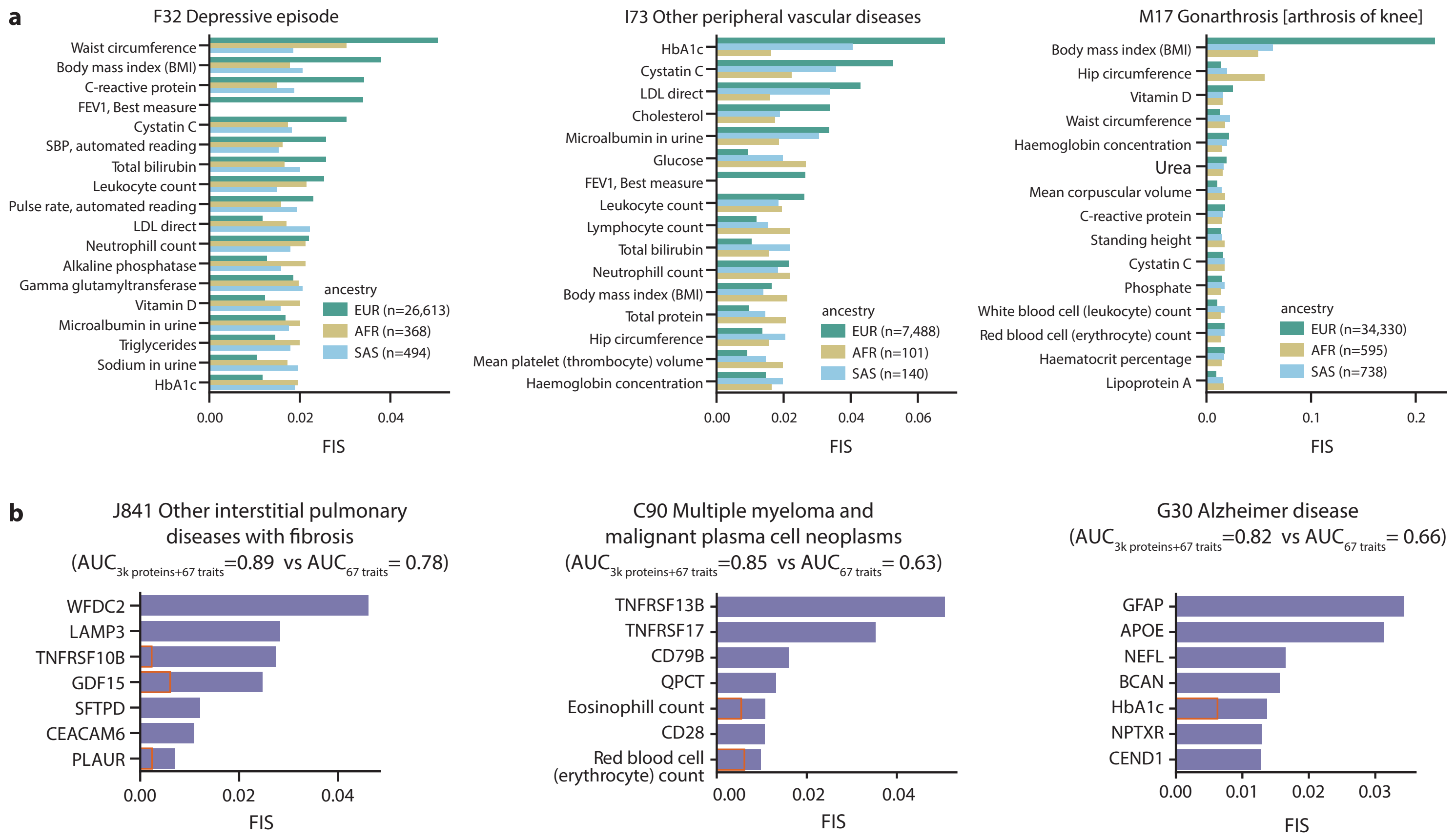

Supplementary Fig. 4 a Bar plots showing top seven features by feature importance scores (FIS) for each ICD10-code (time-agnostic models) and all three ancestries: European (EUR), African (AFR) and South Asian (SAS). Number of UKB individuals diagnosed with given ICD10-code belonging to each ancestry is shown (n). FEV1: forced expiratory volume, SBP: systolic blood pressure, HbA1c: glycated haemoglobin. **b** Bar plots showing top seven features by FIS for each ICD10-code (time-agnostic) models when using UKB Olink 3k proteins + 67 traits along with clinical covariates (see Methods). Median FIS for each feature across all ICD10 codes (time-agnostic models only) is shown in pink bar-plots.

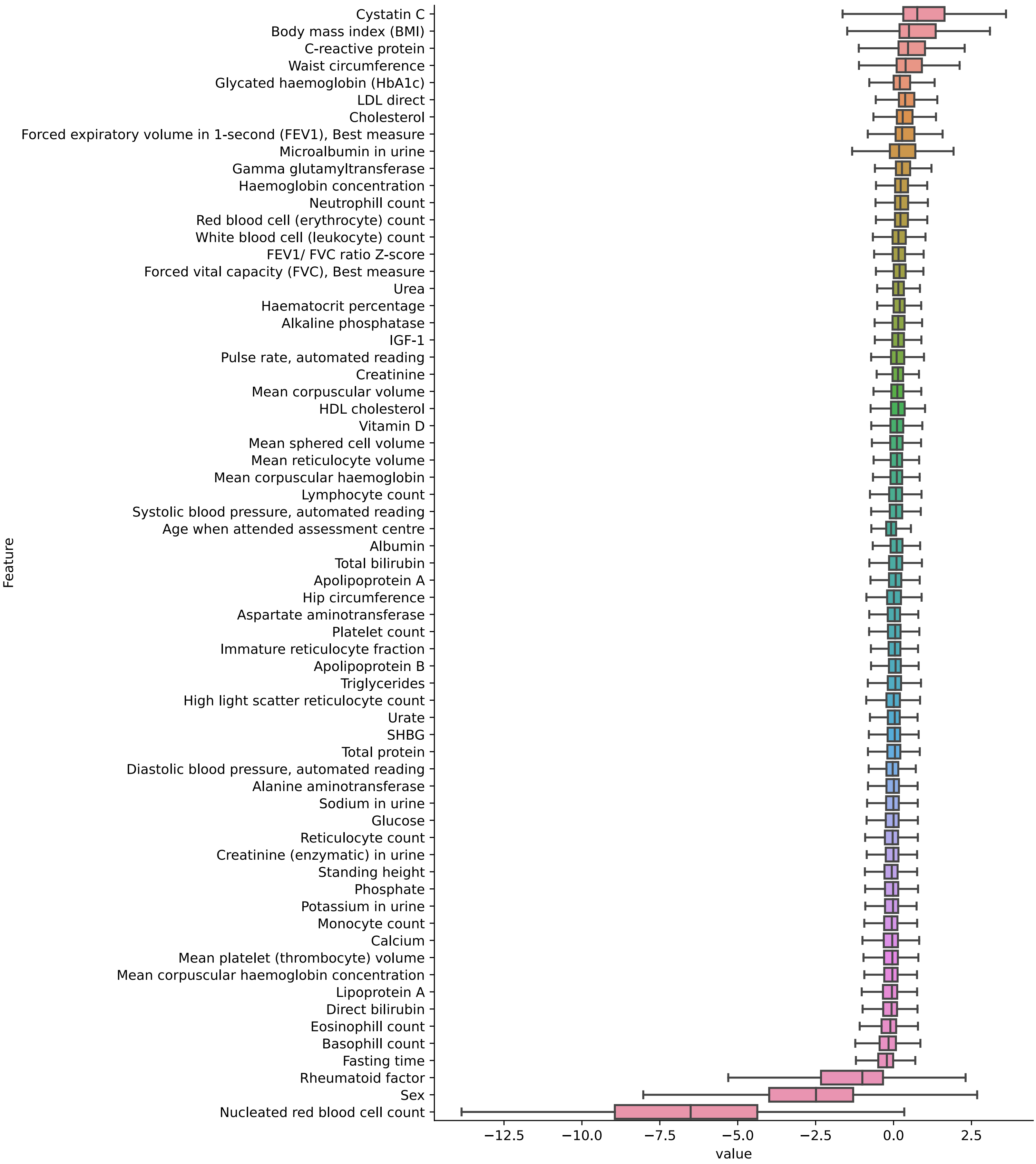

Supplementary Fig. 5 Distribution of normalised feature importance scores across all 3,196 MILTON phenotypes. Each box-plot shows median as centre line, 25th percentile as lower box limit, 75th percentile as upper box-limit, whiskers extend to 25th percentile – 1.5 *interquartile range at the bottom and 75th percentile + 1.5*interquartile range at the top, points denote outliers. Each color denotes the corresponding feature on y-axis. LDL: low density lipoprotein, IGF: insulin-like growth factor, SHBG: sex hormone binding globulin.

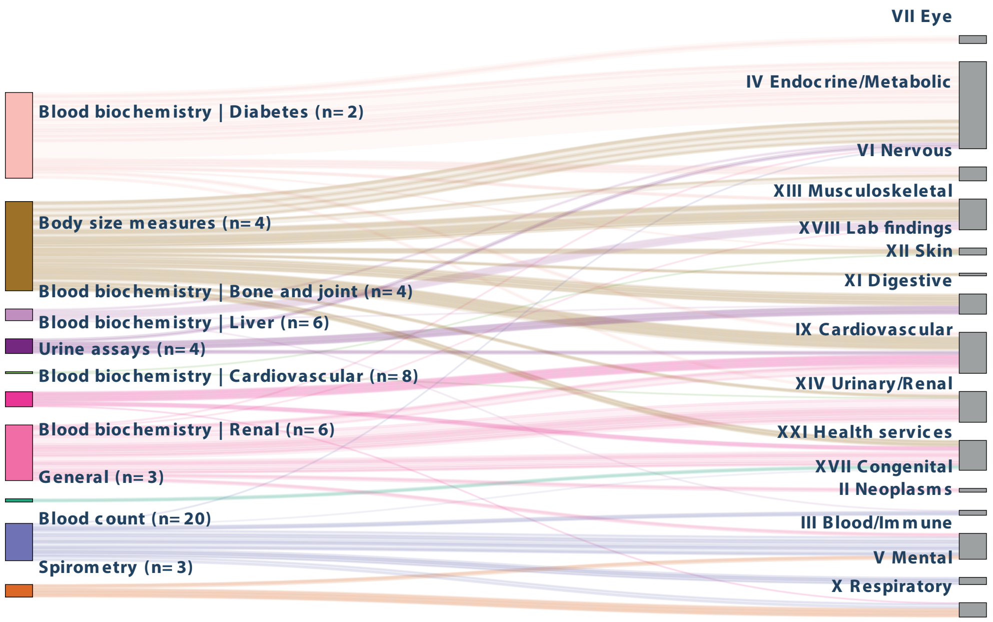

Supplementary Fig. 6. Overview of most important biomarker features learnt by MILTON per ICD10-code: mapping (shown as Sankey plot) of feature categories (left) to ICD10 chapters (right) where each line represents a single feature (with FIS>0.1) mapped to a single ICD10-code. Here, n=number of features in each category and '|' denotes the disease area mapped to the given set of biomarkers. Please note that all four biomarkers derived from urine assays were mapped to 'Renal' disease area.

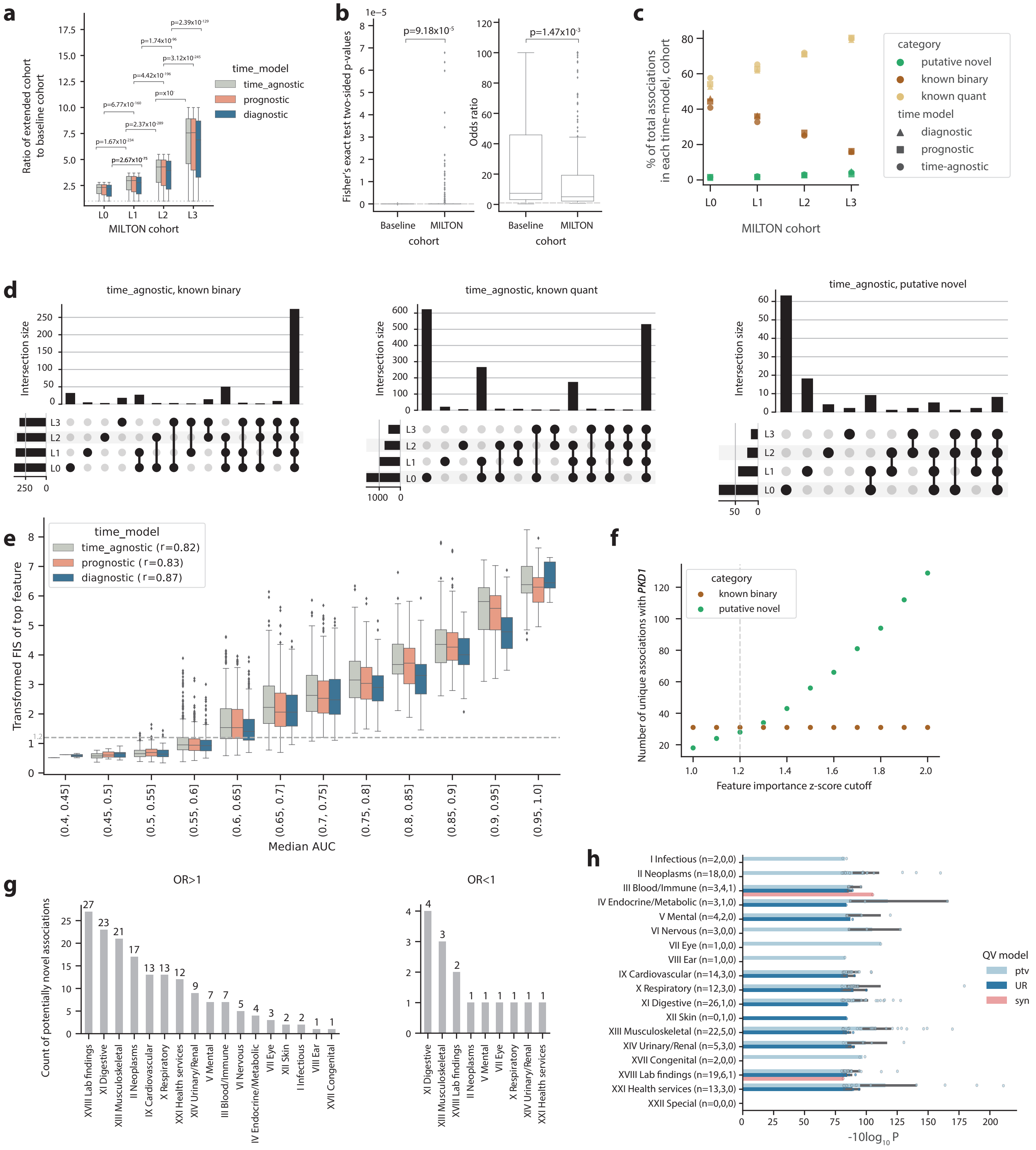

Supplementary Fig. 7 a Box plots comparing the cohort ratio [(number of cases in baseline cohort + number of newly predicted cases)/number of cases in baseline cohort] across each MILTON cohort. Baseline cohort has a cohort ratio of 1. For each cohort on x-axis, results are shown for 3,196 ICD10 codes for time-agnostic model, 2,419 codes for prognostic model and 1,547 codes for diagnostic model. Mann-Whitney U test two-sided p-values are shown. **b** Box plot comparing the p-values (left) and odds ratio (right) of PheWAS performed on baseline cohorts (p<1e-8) with MILTON extended cohorts across 234 unique gene-ICD10 associations. Mann-Whitney U test two-sided p-value are shown. **c** Distribution of known binary ($p_{MILTON}<1x10^{-8}$ & $p_{Baseline}< 1x10^{-8}$), known quant ($p_{MILTON}<1x10^{-8}$ & $p_{Baseline}> 1x10^{-8}$ & $p_{quant\ PheWAS\ for\ FIS>1.2}<1x10^{-8}$) and putative novel ($p_{MILTON}<1x10^{-8}$ & $p_{Baseline}> 1x10^{-8}$ & $p_{quant\ PheWAS\ for\ FIS>1.2}>1x10^{-8}$) associations across multiple MILTON augmented cohorts and time-models. Here, two-sided Fisher's exact test was performed to calculate p-values for each ICD10, cohort, gene, QV model and time model. No muliple testing correction was applied. **d** UpSet plots showing the distribution of known binary, known quant and putative novel gene-ICD10 associations across different MILTON extended cohorts for time-agnostic model. **e** Box plot comparing the transformed FIS of top feature per ICD10-code with the median AUC value of that ICD10-code. Dotted line denotes FIS z-score cutoff. Pearson's correlation coefficient (r) is indicated in brackets in-front of each time-model in the legend. **f** Scatter plots showing the number of unique ICD10 codes associated with *PKD1* ($p<1x10^{-8}$) at different feature importance z-score cutoffs. **g** Bar plot showing the number of unique gene-ICD10 associations per chapter. **h** Distribution of scores ($-10log_{10}P$) from putative novel gene-disease associations by chapter and across 3 out of the 11 QV models employed by MILTON: protein truncating variant (ptv; n=148), ultra rare variant (UR; n=32), synonymous variant (syn; n=2). P-values are from Fisher's exact test (two-sided, unadjusted). More details about the models can be found in Wang et al. 2021. Error-bars denote 95% confidence intervals and end of the bars represent mean scores per chapter. In panels **a**, **b** and **e**, each box-plot shows median as centre line, 25th percentile as lower box limit, 75th percentile as upper box-limit, whiskers extend to 25th percentile – 1.5*interquartile range at the bottom and 75th percentile +1.5*interquartile range at the top, points denote outliers.

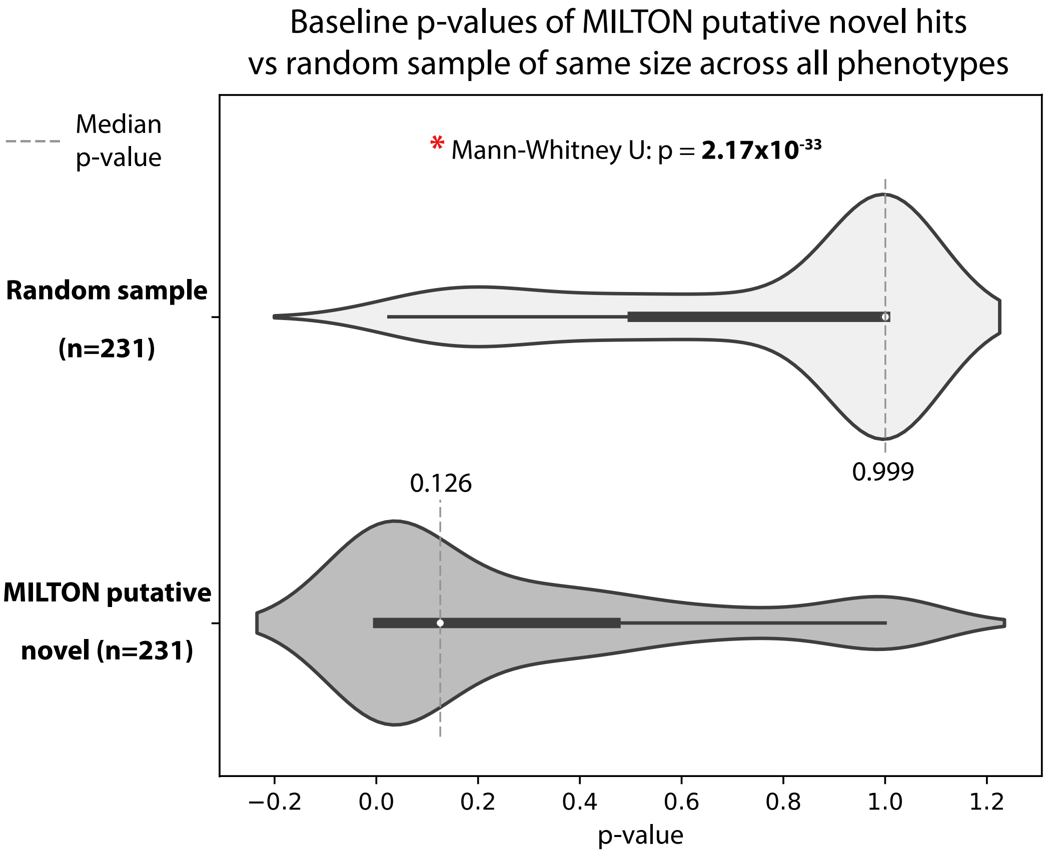

Supplementary Fig. 8 Comparison of baseline p-values of putative novel hits derived from performing PheWAS on MILTON extended cohorts with randomly selected genes of same size. Mann Whitney two-sided p-values are shown on the top.
Each box-plot shows median as centre line, 25th percentile as lower box limit, 75th percentile as upper box-limit, whiskers extend to 25th percentile – 1.5 * interquartile range at the bottom and 75th percentile + 1.5*interquartile range at the top, points denote outliers.

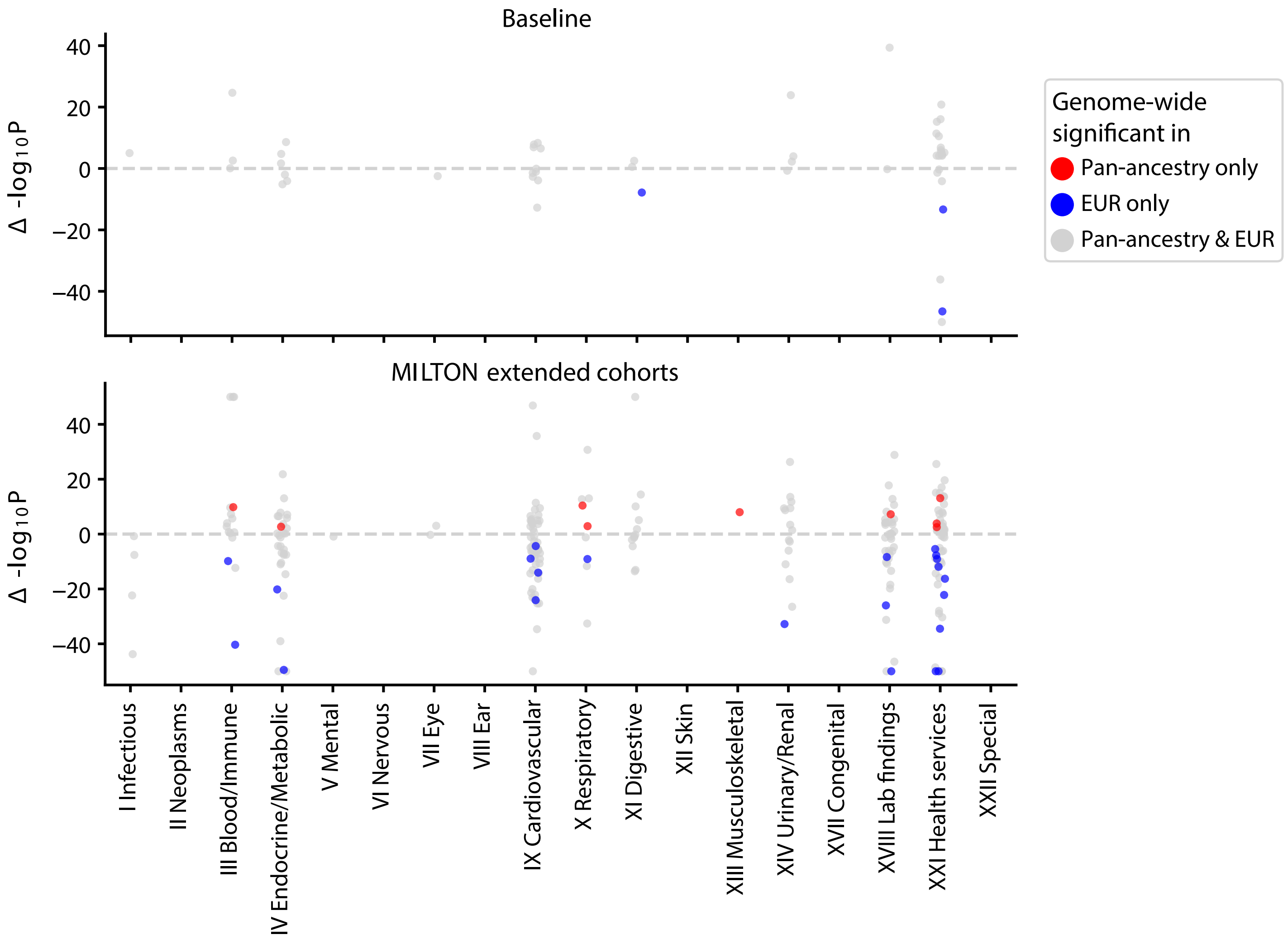

Supplementary Fig. 9 Scatter plot showing change in scores ($-10\log_{0}$ P) between the pan-ancestry and European (EUR) only analysis across 197 ICD10-codes that acheived AUC>0.6 in at least 2 ancestries. Only unique gene (with protein truncating variants)-ICD10 associations that acheived genome-wide significance ($p<1x10^{-8}$) in either or both of the analysis and had non-null score difference are shown: 50 associations in baseline cohort and 238 associations in MILTON extended cohorts. Scores are capped at ±50. P-values are from Fisher's exact test (two-sided, unadjusted).

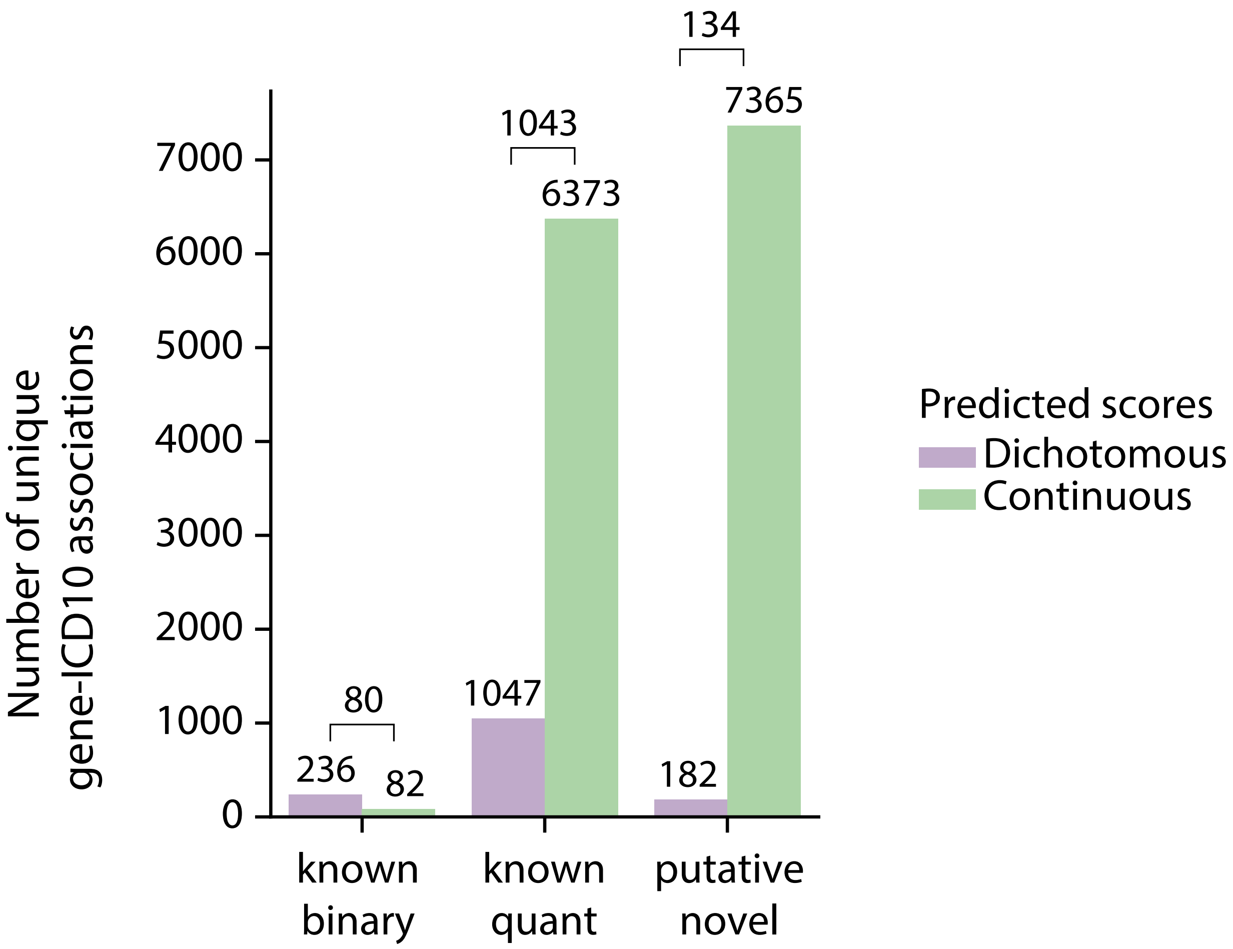

Supplementary Fig. 10 Bar plots comparing number of gene-ICD10 associations obtained when using dichtomous cohorts vs predicted probability scores as continuous phenotype. Numbers above brackets show common associations obtained from both these two approaches.

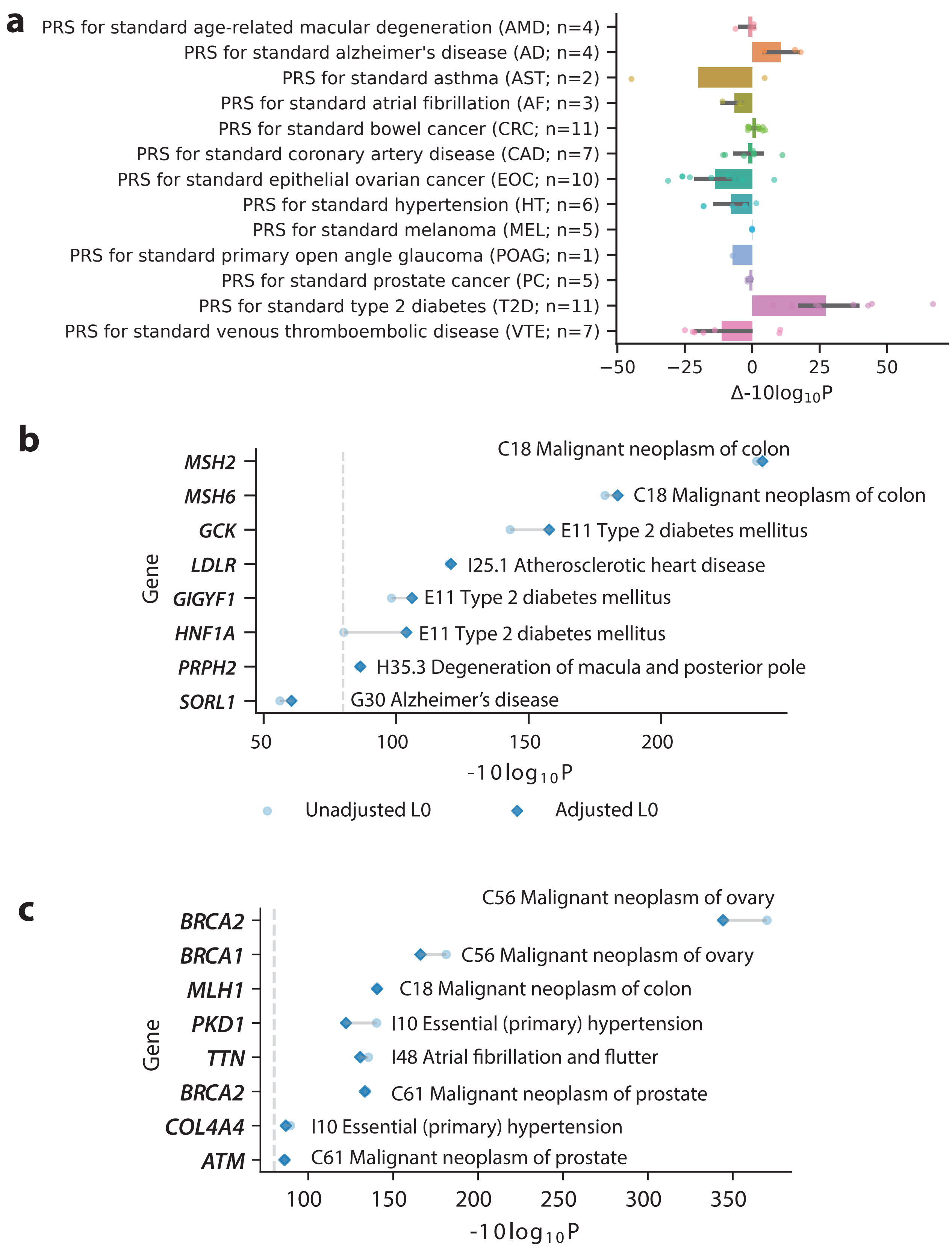

Supplementary Fig. 11 a Bar plots showing difference between scores (-10log$_{10}$P) when running PheWAS on adjusted cohorts vs unadjuated cohorts. Each bar denotes mean statistic with error bars representing 95% confidence interval. Color denote a PRS. **b** Dumble plots showing baseline associations that got boost in scores when using adjusted cohorts. **c** Dumble plots showing baseline associations that got boost in scores when using unadjusted cohorts. Grey line in panels **b** and **c** denote the score corresponding to genome-wide significance threshold (p=1x10^{-8}). P-values are from Fisher's exact test (two-sided, unadjusted).

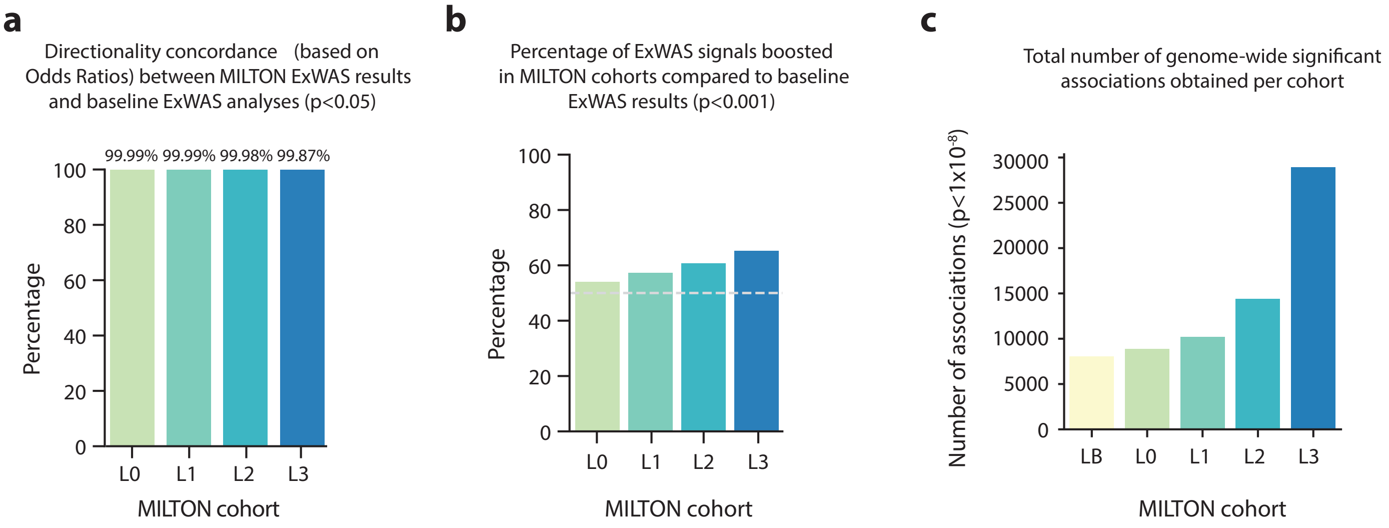

Supplementary Fig. 12 Summary resuls for exome-wide association studies (ExWAS) using allelic model (Quanli et al. 2021). **a** Barplots show percentage concordance of direction of effect between baseline variant-ICD10 associations ($p<0.05$) and those observed in MILTON extended cohorts ($p<0.05$). **b** Barplots showing percentage of baseline associations ($p<0.001$) that acheived a boost in signal ($p_{MILTON} < p_{Baseline}$) when ExWAS was repeated on each of the MILTON extended cohorts. **c** Total number of genome-wide significant associations obtained per cohort. "LB" denotes baseline cohort for each phenotype. In panels **a**-**c**, Fisher's exact test two-sided p-values are reported and no adjustment for multiple testing was made.

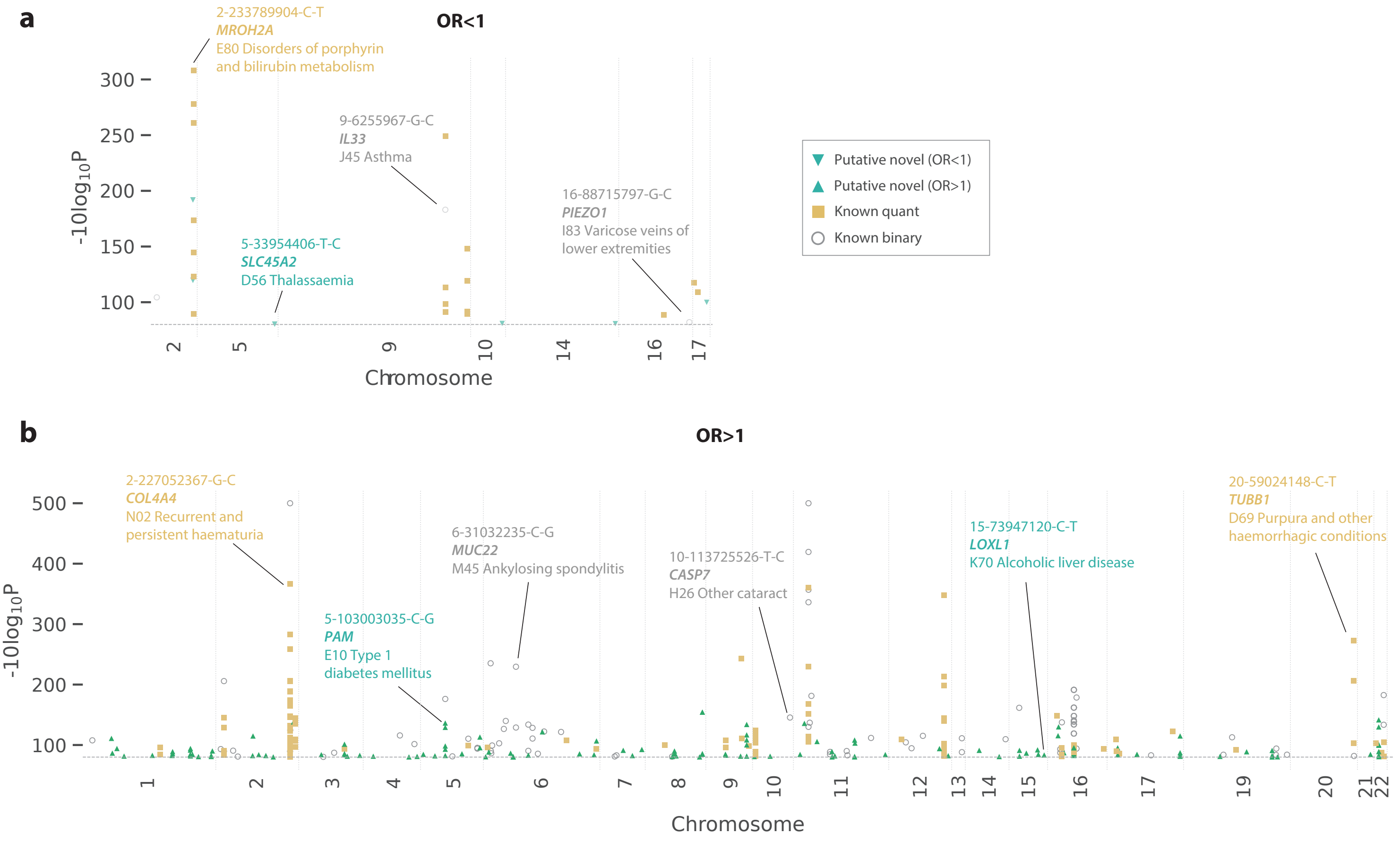

Supplementary Fig. 13 Manhattan plots showing distribution of rare variant (minor allele frequency < 0.01) - ICD10 associations ($p<1\times10^{-8}$) obtained from ExWAS using allelic model (Quanli et al. 2021). **a** Associations with odds ratio (OR) < 1. **b** Associations with OR>1. Please note that labels include variant name on the top as chromosome - position - reference allele - alternate allele, gene name in second line and ICD10 code + description in remaining lines. P-values are from Fisher's exact test (two-sided, unadjusted).

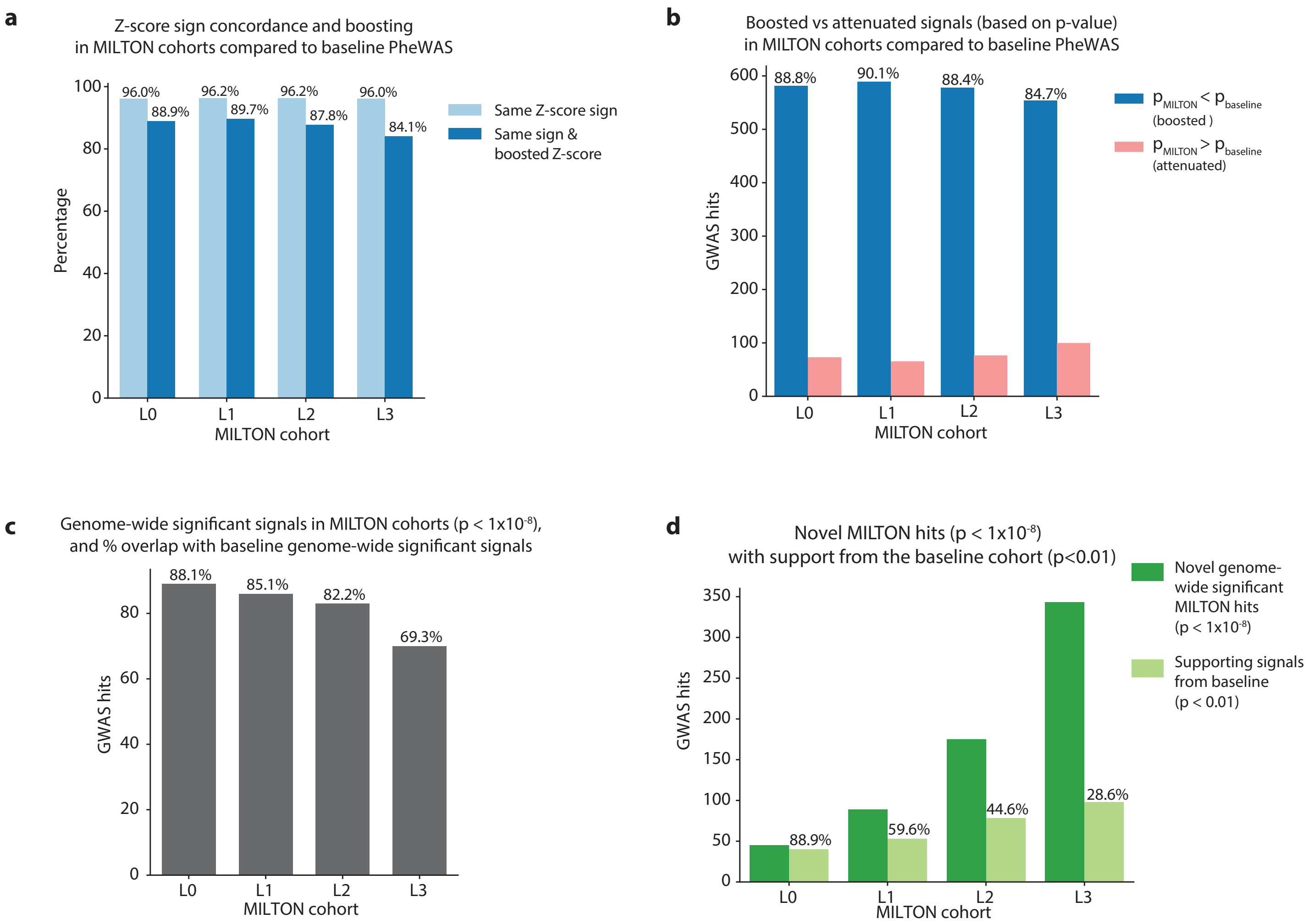

Supplementary Fig. 14 Summary results from common variant genome-wide association studies (GWAS) for 20 phenotypes. **a** Bar plot showing percentage concordance in direction of Z-scores and any observed increase in Z-scores between baseline cohorts and MILTON extended cohorts. **b** Bar plot comparing percentage increase or decrease in p-values between baseline cohorts and MILTON extended cohorts. **c** Bar plot showing the number of baseline associations ($p<1x10^{-8}$) recovered in each of the MILTON cohorts. **d** Bar plot showing the number of putative novel associations in each cohort that were also significant in baseline cohort (p<0.001). P-values are derived from REGENIE-Firth regression GWAS (two-sided, unadjusted).

GWAS summary results for 19 phenotypes (E11 excluded)

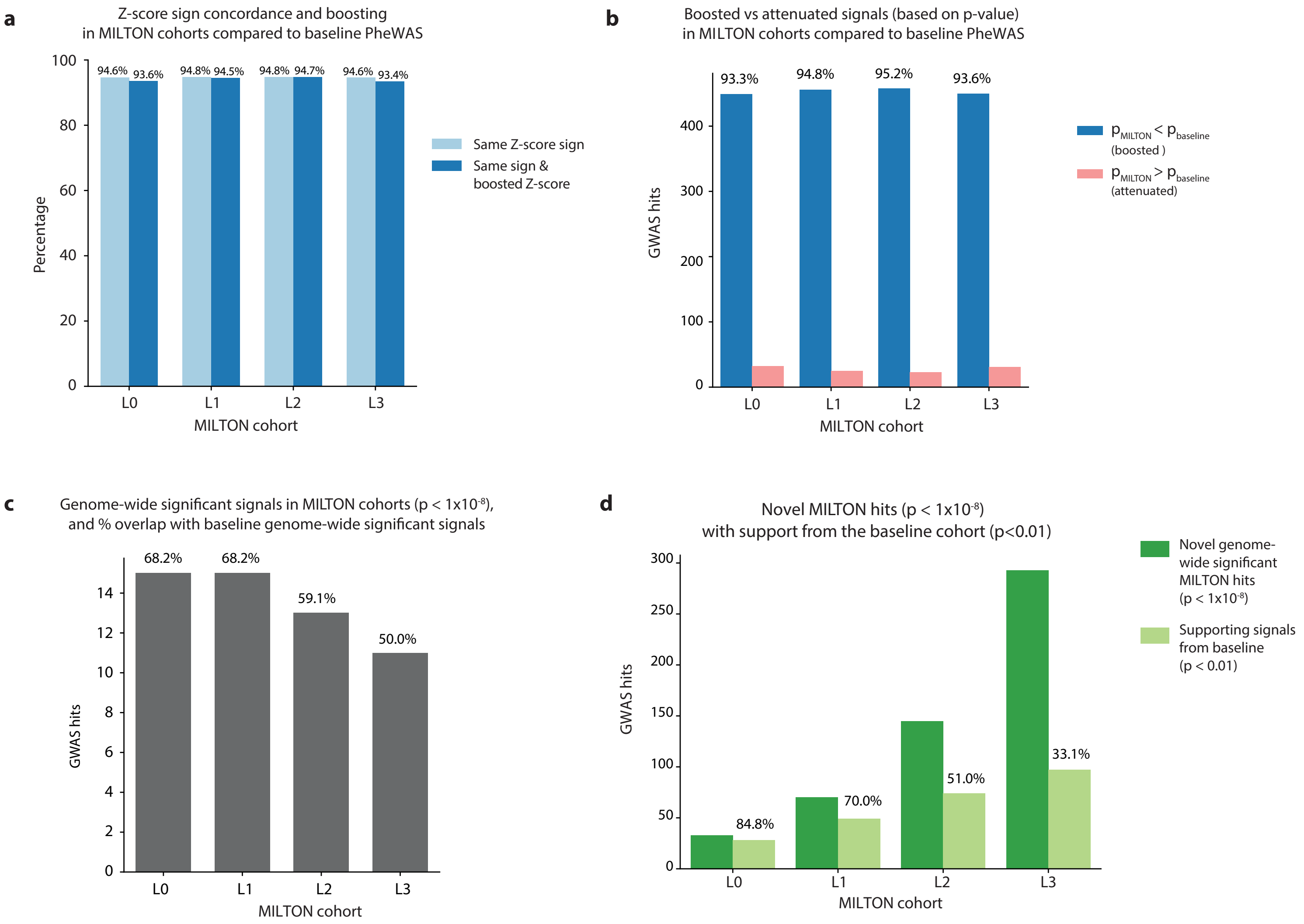

Supplementary Fig. 15. Summary results from common variant genome-wide association studies (GWAS) for all phenotypes except E11 insulin independent diabetes mellitus. **a** Bar plot showing percentage concordance in direction of Z-scores and any observed increase in Z-scores between baseline cohorts and MILTON extended cohorts. **b** Bar plot comparing percentage increase or decrease in p-values between baseline cohorts and MILTON extended cohorts. **c** Bar plot showing the number of baseline associations ($p<1x10^{-8}$) recovered in each of the MILTON cohorts. **d** Bar plot showing the number of putative novel associations in each cohort that were also significant in baseline cohort (p<0.001). P-values are derived from REGENIE-Firth regression GWAS (two-sided, unadjusted).

GWAS on 20 ICD10 codes

- Comparison of observed vs theoretical Z-score gains from GWAS analysis **across 20 ICD10 codes**
- Num. of unique common variants: **654**
- Fitted lines represent the **average** observed or theoretical values across all 20 examined ICD10 codes

Linear fit of "known Z" vs "**observed Z**"

Linear fit of "known Z" vs "**theoretical Z**"

Equivalence line (y=x)

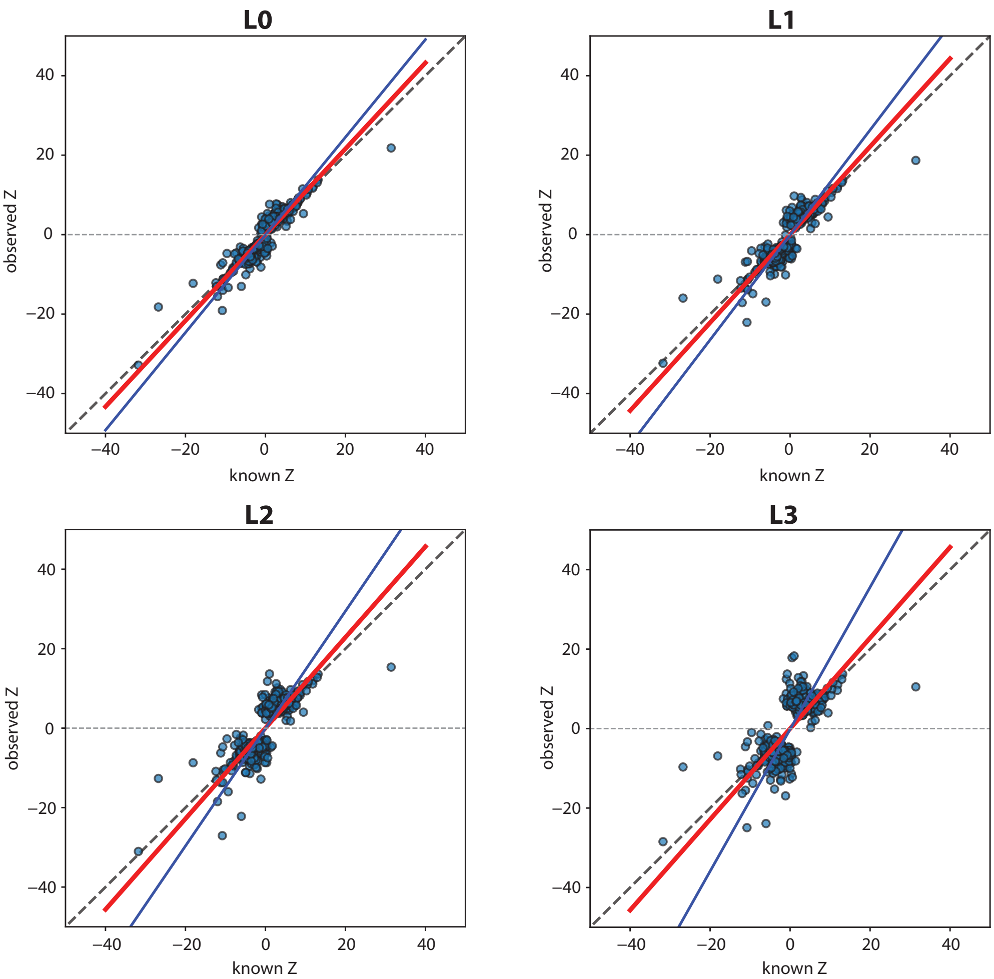

Supplementary Figure 16. Comparison of observed Z scores in MILTON extended cohorts (L0-L3) vs theoretical Z gains (i.e. if our case/control classifications were perfect) with the increased sample sizes across 20 ICD10 codes, analysed for common variants.

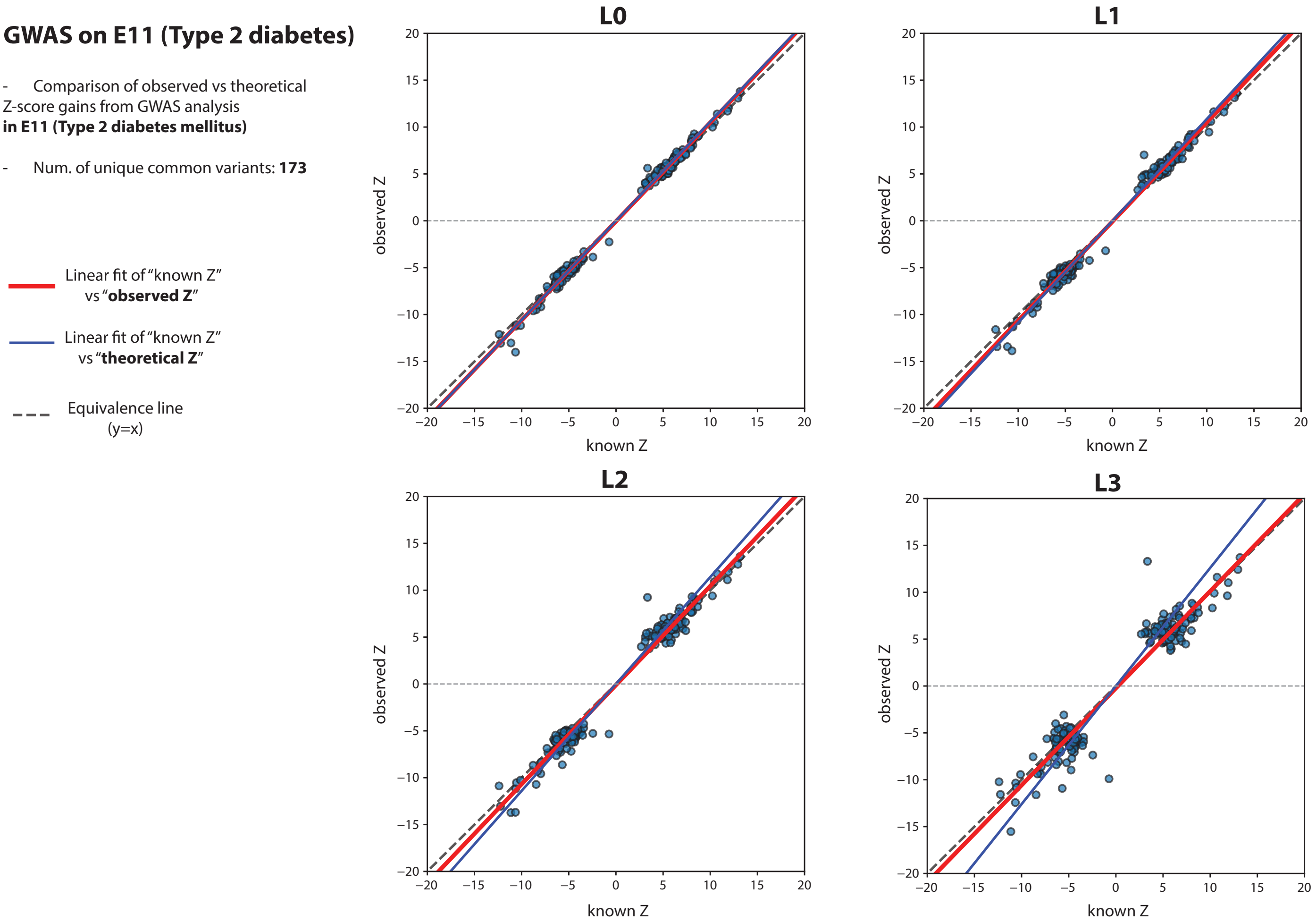

Supplementary Figure 17. Comparison of observed Z scores in MILTON extended cohorts (L0-L3) vs theoretical Z gains (i.e. if our case/control classifications were perfect) with the increased sample sizes for the E11 ICD10 code, analysed for common variants.

GWAS on E16 (Other disorders of pancreatic internal secretion)

- Comparison of observed vs theoretical Z-score gains from GWAS analysis **in E16 (Other disorders of pancreatic internal secretion)**

- Num. of unique common variants: **70**

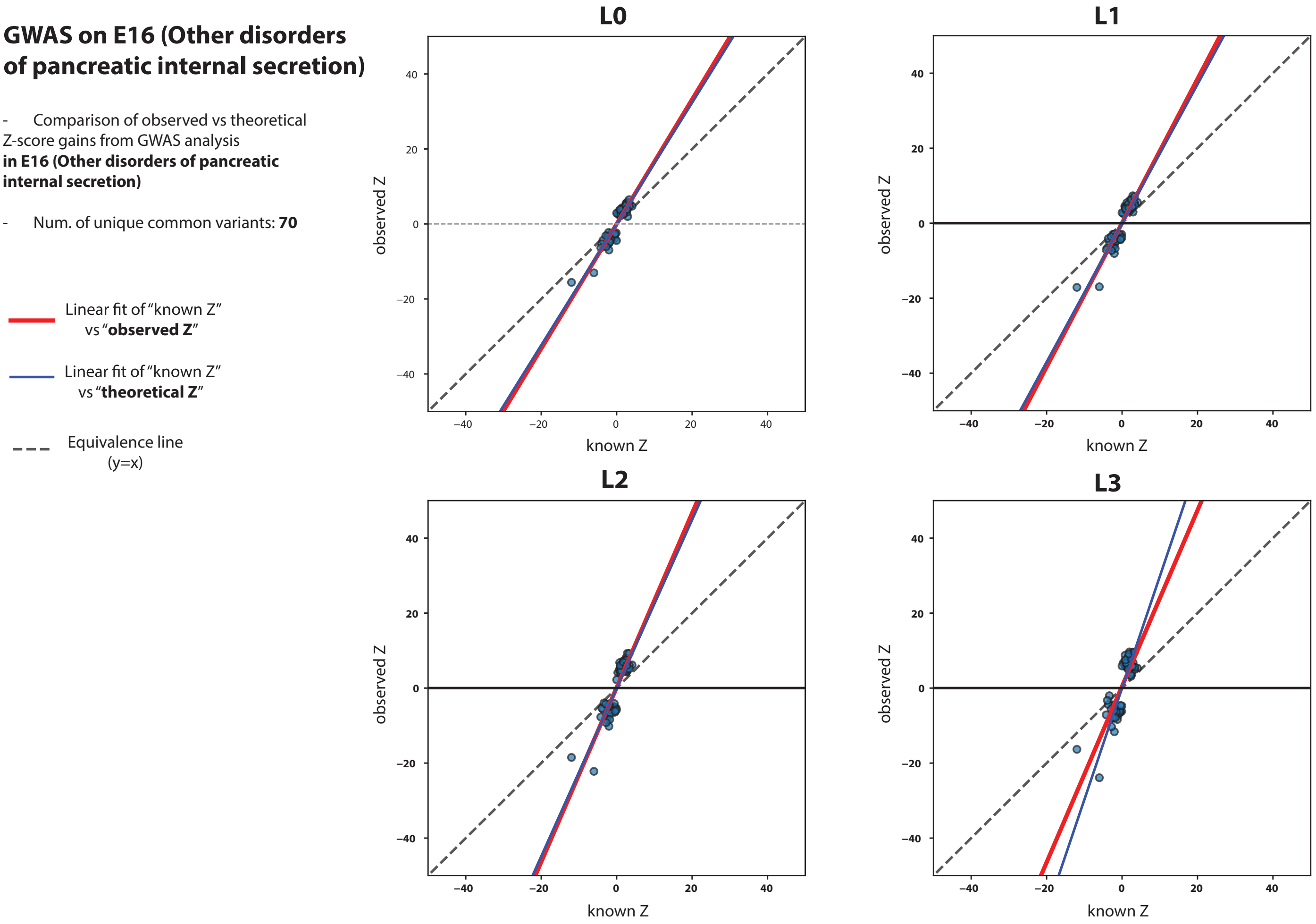

Supplementary Figure 18. Comparison of observed Z scores in MILTON extended cohorts (L0-L3) vs theoretical Z gains (i.e. if our case/control classifications were perfect) with the increased sample sizes for the E16 ICD10 code, analysed for common variants.

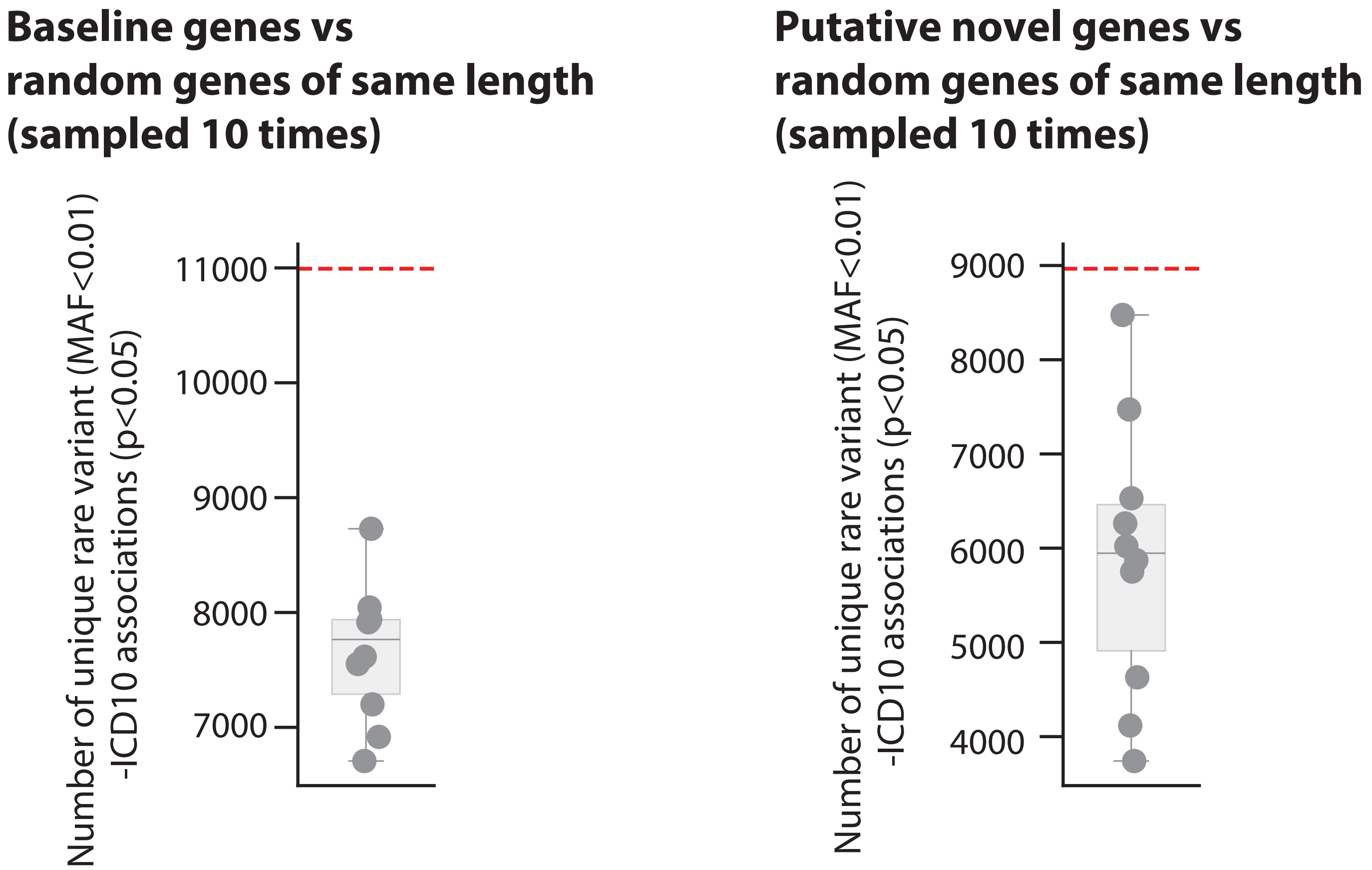

Supplementary Fig. 19 Boxplots comparing the number of unique rare-variant-ICD10 associations (p<0.05) fetched from FinnGen when using baseline (left) or putative novel (right) gene-ICD10 associations vs using random genes of same length. Red dashed line represents the number of unique variant-ICD10 associations (p<0.05) retreived when using baseline (left) or putative novel (right) gene-ICD10 associations. All variants (MAF<0.001) associated with given ICD10 code (p<0.05) that had given gene listed in the "nearest_gene" column was fetched for this analysis. Here, FinnGen p-values are derived from GWAS SAIGE (two-sided, unadjusted). Each box-plot shows median as centre line, 25th percentile as lower box limit, 75th percentile as upper box-limit, whiskers extend to 25th percentile – 1.5* interquartile range at the bottom and 75th percentile + 1.5*interquartile range at the top, points denote outliers.

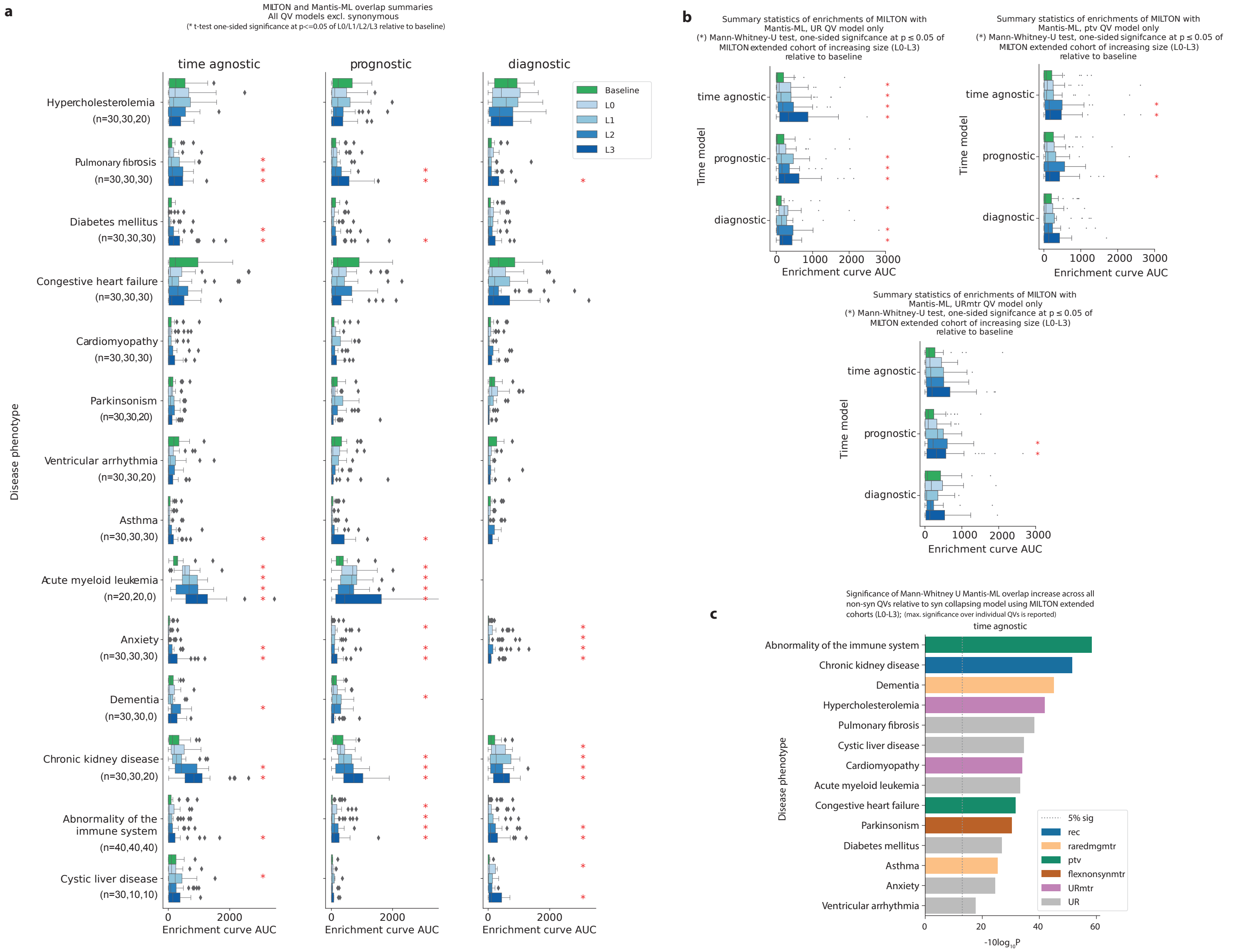

Supplementary Fig. 20 Enrichment of high-ranking MANTIS-ML genes in MILTON extended cohorts (PheWAS p<0.05) compared to baseline cohorts. **a** Per disease-area and non-synonymous QV models. **b** All disease areas and UR, URmtr and ptv QV models: n=42 for time agnostic, n=31 for diagnostic and n=40 for prognostic. More details about these QV models can be found in Wang et al. 2021[2]. In panels a and b, each box-plot shows median as centre line, 25th percentile as lower box limit, 75th percentile as upper box-limit, whiskers extend to 25th percentile – 1.5 * interquartile range at the bottom and 75th percentile + 1.5*interquartile range at the top, points denote outliers. **c** Bar plot showing the QV model per disease area that achieved the highest score (-10log10P) over synonymous QV model. In panels **a** and **b**, no multiple testing correction was performed.

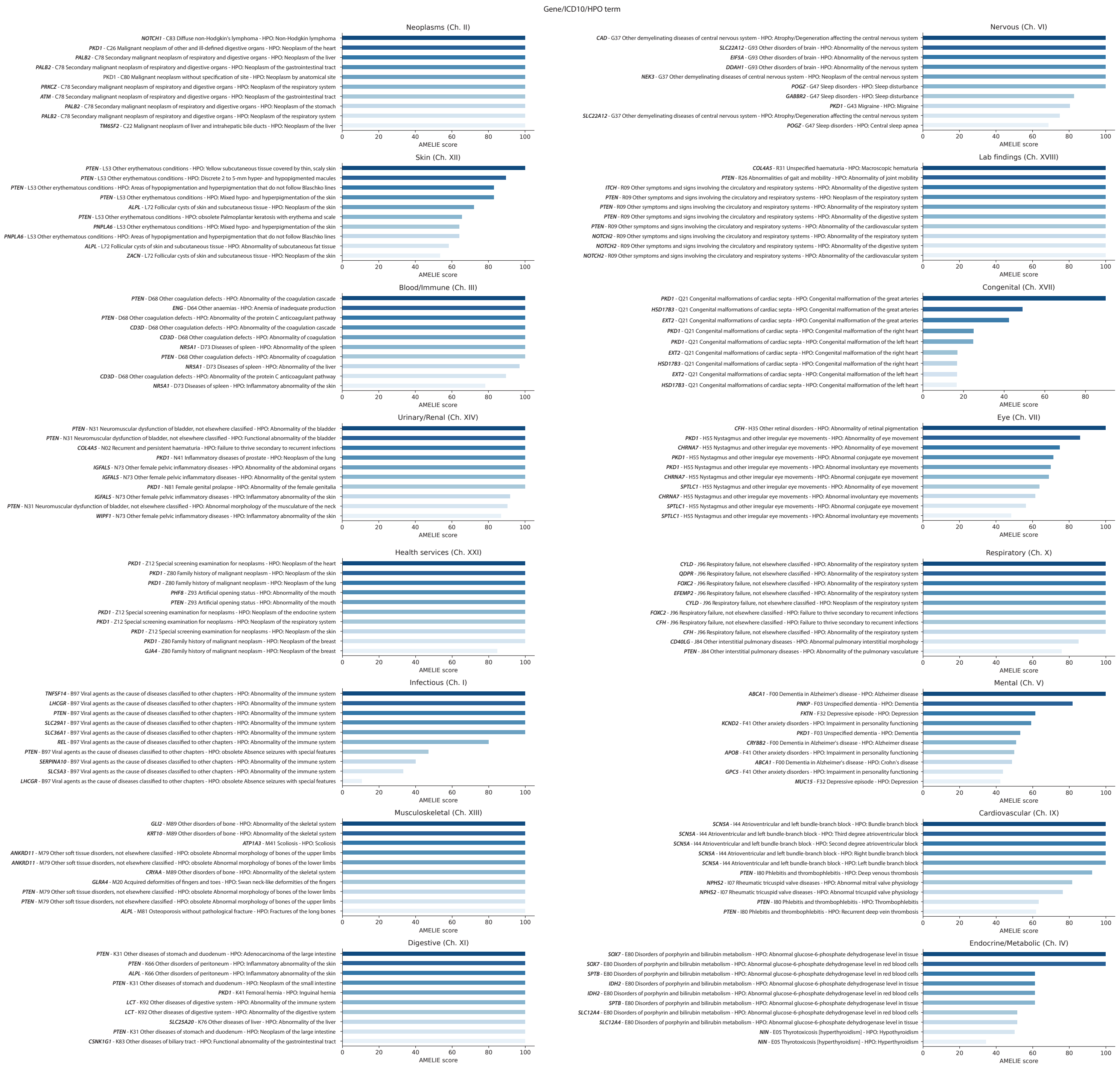

Supplementary Fig. 21 Bar plots showing top 10 phenotypes by AMELIE enrichment scores (x-axis) for each chapter. For each y-axis label, putative novel gene-ICD10 (at 3-character level) association is shown followed by one of the 5 HPO terms this ICD10 term was mapped to using natural language processing. Darker blue shades represent higher AMELIE score and vice versa. Ch. denotes chapter.

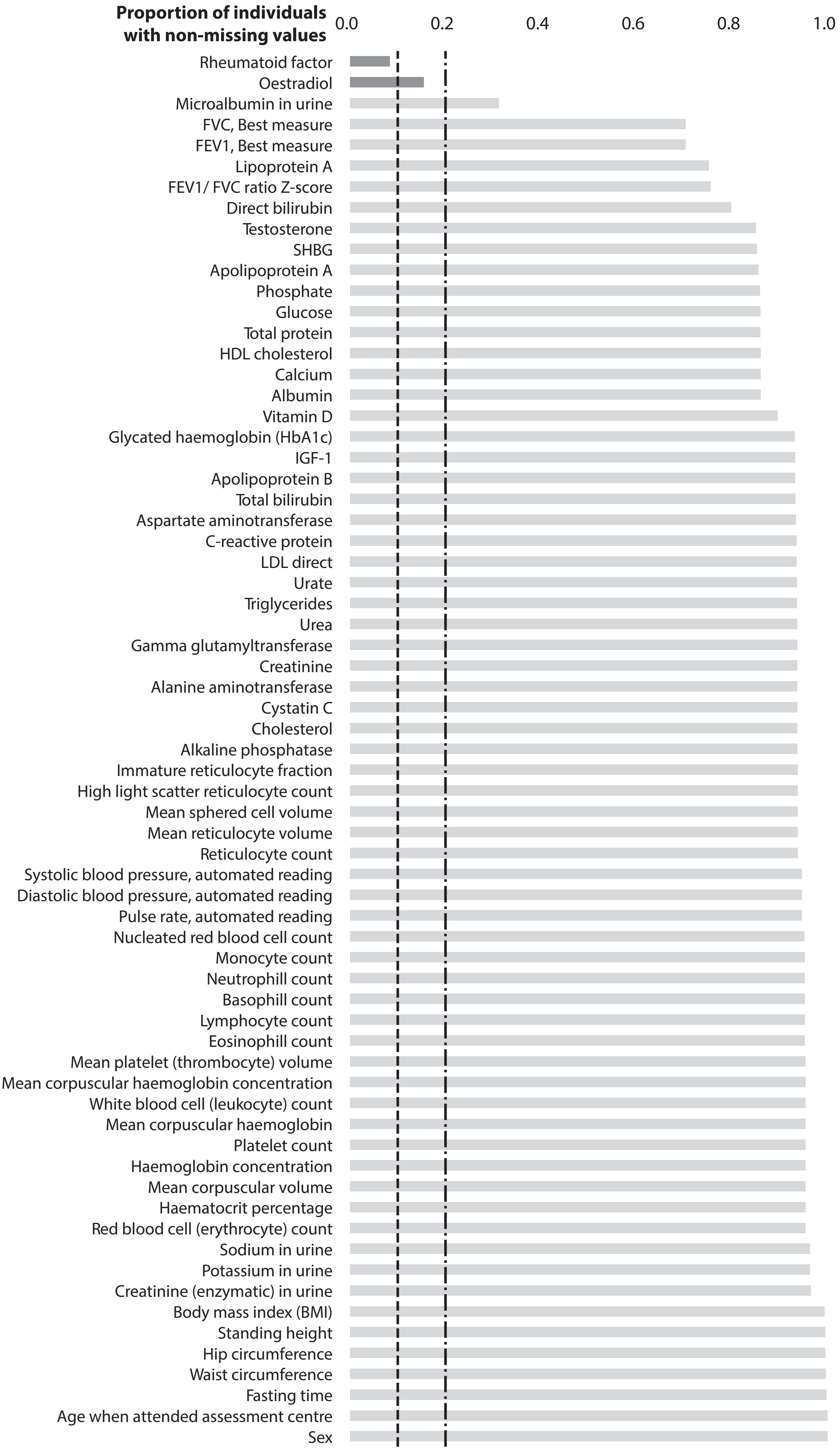

Supplementary Fig. 22 Bar plot showing the proportion (top) of UKB individuals with non-missing values for a given feature (left). Dark gray bars indicate features with greater than 80% missing values. FVC: Forced vital capacity; FEV1: Forced expiratory volume in 1-second; LDL: low density lipoprotein; IGF: insulin-like growth factor; SHBG: sex hornome binding globulin.

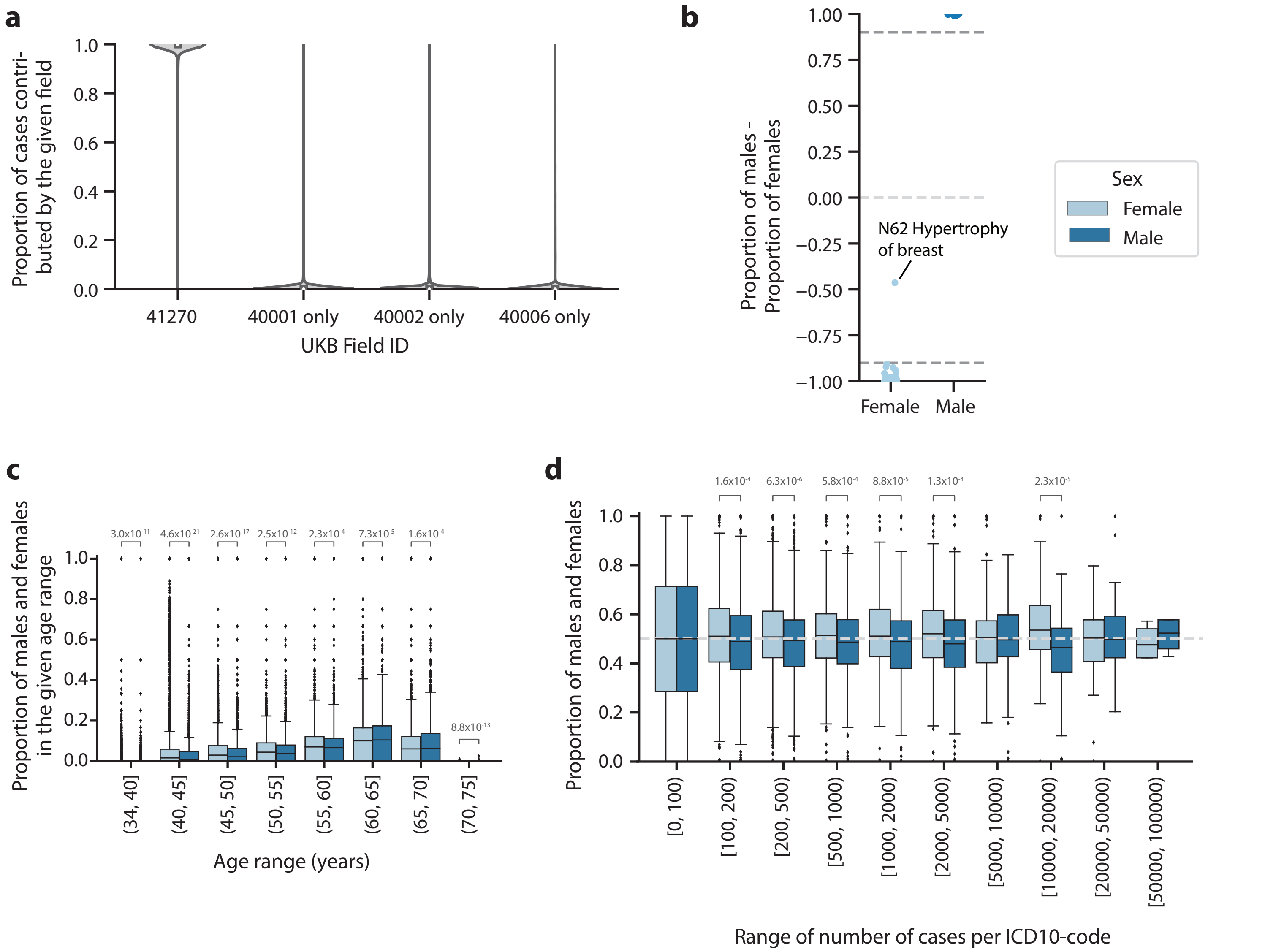

Supplementary Fig. 23 a Violin plot showing the proportion of diagnosed cases contributed by UKB fields 41270: hospital inpatient diagnosis, 40001: underlying (primary) cause of death, 40002: contributory (secondary) causes of death and 40006: Cancer registry. **b** Scatter plot showing the difference in proportion of males and females in sex-specific diseases with value of –1 expected for female-specific diseases and value of 1 expected for male-specific diseases. Dotted lines indicate proportion of ±0.1. **c** Boxplots showing the distribution of males and females in different age-ranges (n=13,942 ICD10 codes). **d** Boxplots showing the distribution of males and females across ICD10 codes with increasing number of diagnosed cases ($n_{0\text{-}100}$=10,378; $n_{100\text{-}200}$=937; $n_{200\text{-}300}$=1,020; $n_{500\text{-}1000}$=567; $n_{1000\text{-}2000}$=412; $n_{2000\text{-}5000}$=329; $n_{5000\text{-}10000}$=148; $n_{10000\text{-}20000}$=90; $n_{20000\text{-}50000}$=56; $n_{50000\text{-}100000}$=4). Mann-Whitney U test, two-sided p-values are shown in panels **c** and **d**. In panels **c** and **d**, each box-plot shows median as centre line, 25th percentile as lower box limit, 75th percentile as upper box-limit, whiskers extend to 25th percentile – 1.5 * interquartile range at the bottom and 75th percentile + 1.5*interquartile range at the top, points denote outliers.

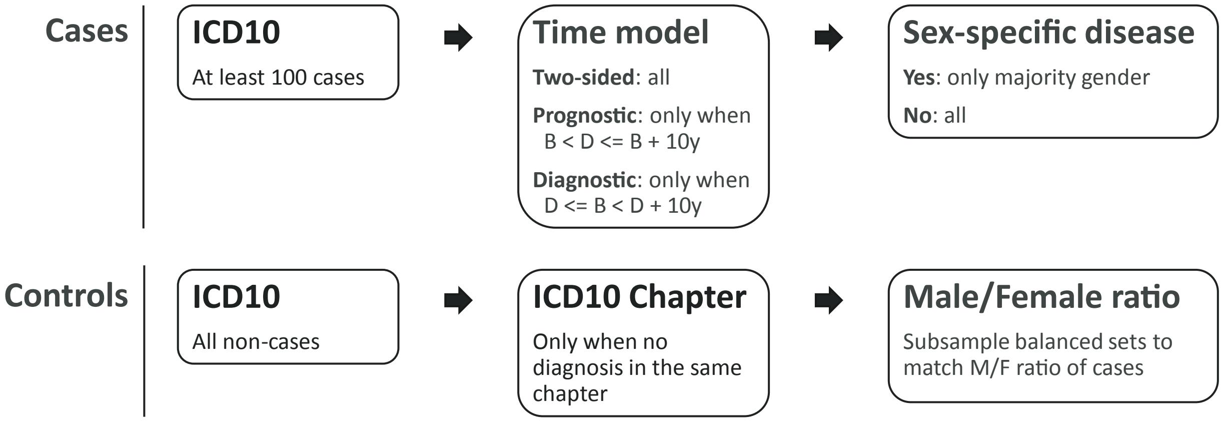

Supplementary Fig. 24 A schematic showing the filtering process for cases and controls: each box is a filter with a condition for accepting samples, B and D stand for biomarker collection and diagnosis dates respectively. The letter "y" denotes number of years between biomarker collection dates and diagnosis dates.

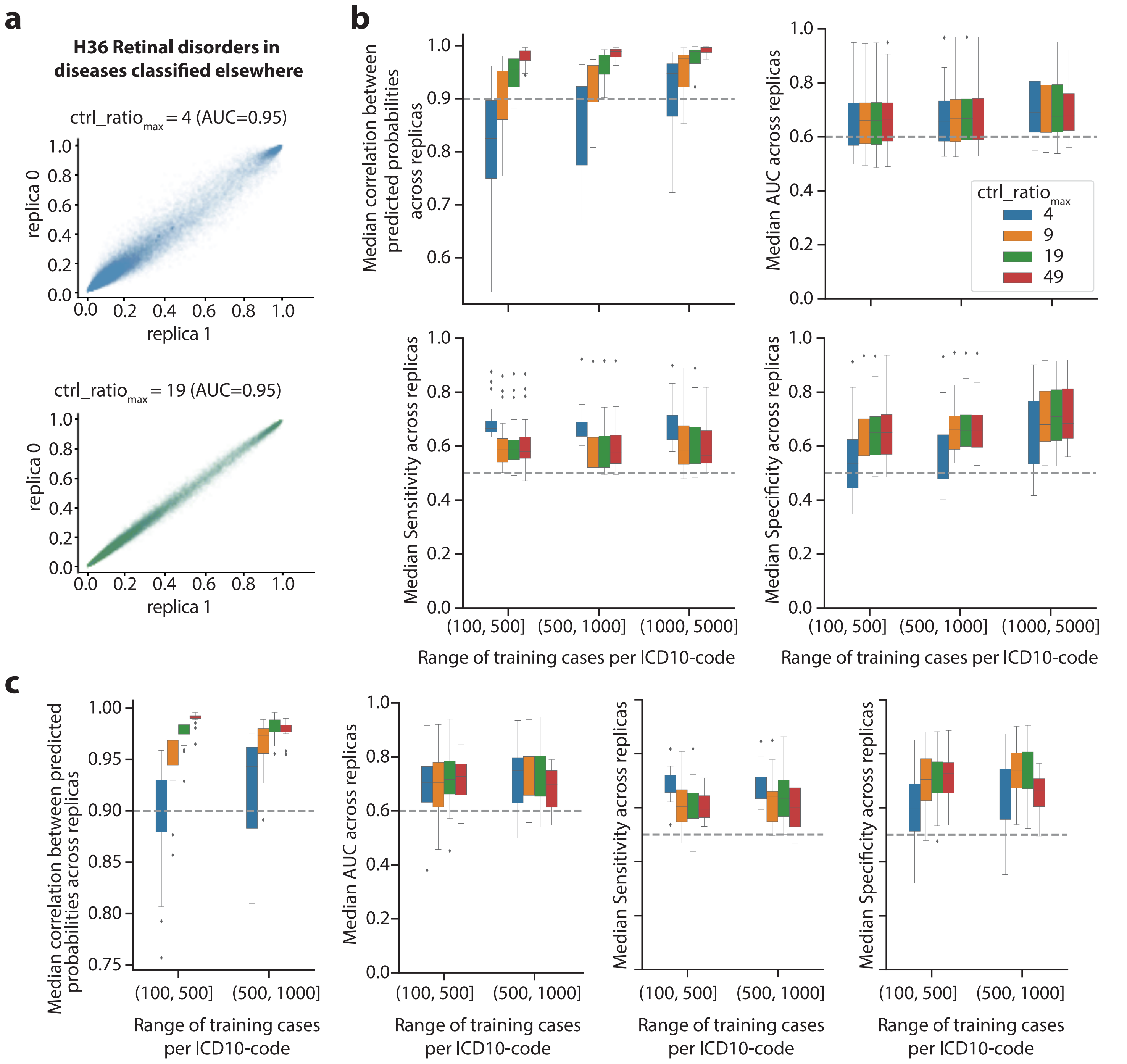

Supplementary Fig. 25 Rationale behind chossing a control ratio (ctrl_ratio) of 19 when sample sizes are high and 9 when less data is available for analysis, such as those for non-European ancestries and Olink proteomics data. **a** Scatter plots comparing probability scores predicted by two of ten replicas in MILTON run for H36. We observe that for ctrl_ratio=4, there is less concordance between scores, especially for individuals with scores roughly around 0.4-0.8. Increasing ctrl_ratio helped to acheived better concordance. **b** Box plots showing the median Pearson's correlation coefficient, AUC, sensitivity and specificity across 10 replicates for each ICD10-code and ctrl-ratio (European ancestry and 67 traits). **c** Same analysis as **b** but using Olink 3k proteins as features and retricting to only those subjects who have this data available. In panels **b** and **c**, each box-plot shows median as centre line, 25th percentile as lower box limit, 75th percentile as upper box-limit, whiskers extend to 25th percentile – 1.5 * interquartile range at the bottom and 75th percentile + 1.5*interquartile range at the top, points denote outliers.

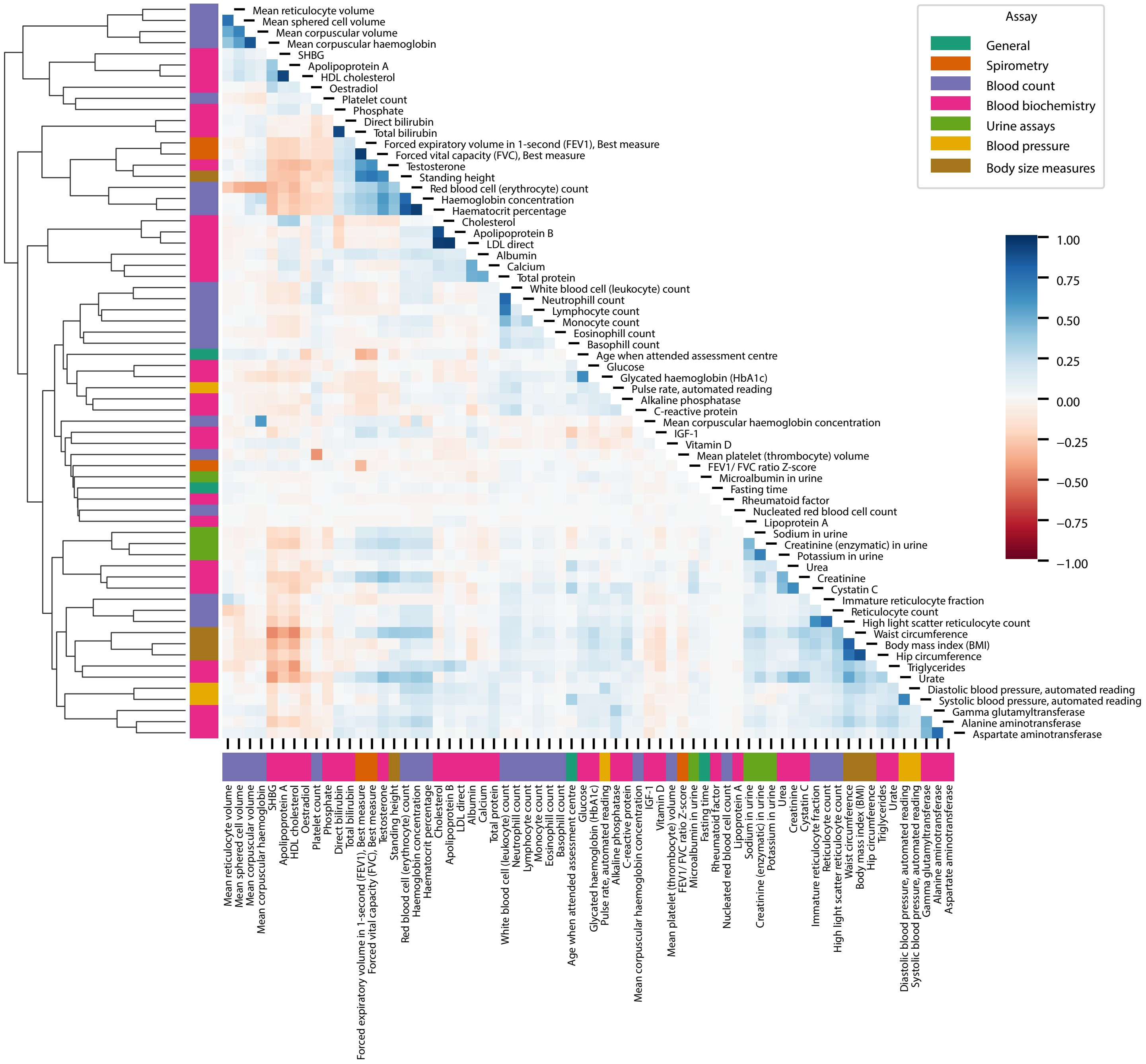

Supplementary Fig. 26 Heatmap showing Pearson's correlation coefficient between non-missing values in features. LDL: low density lipoprotein; IGF: insulin-like growth factor; SHBG: sex hornome binding globulin.

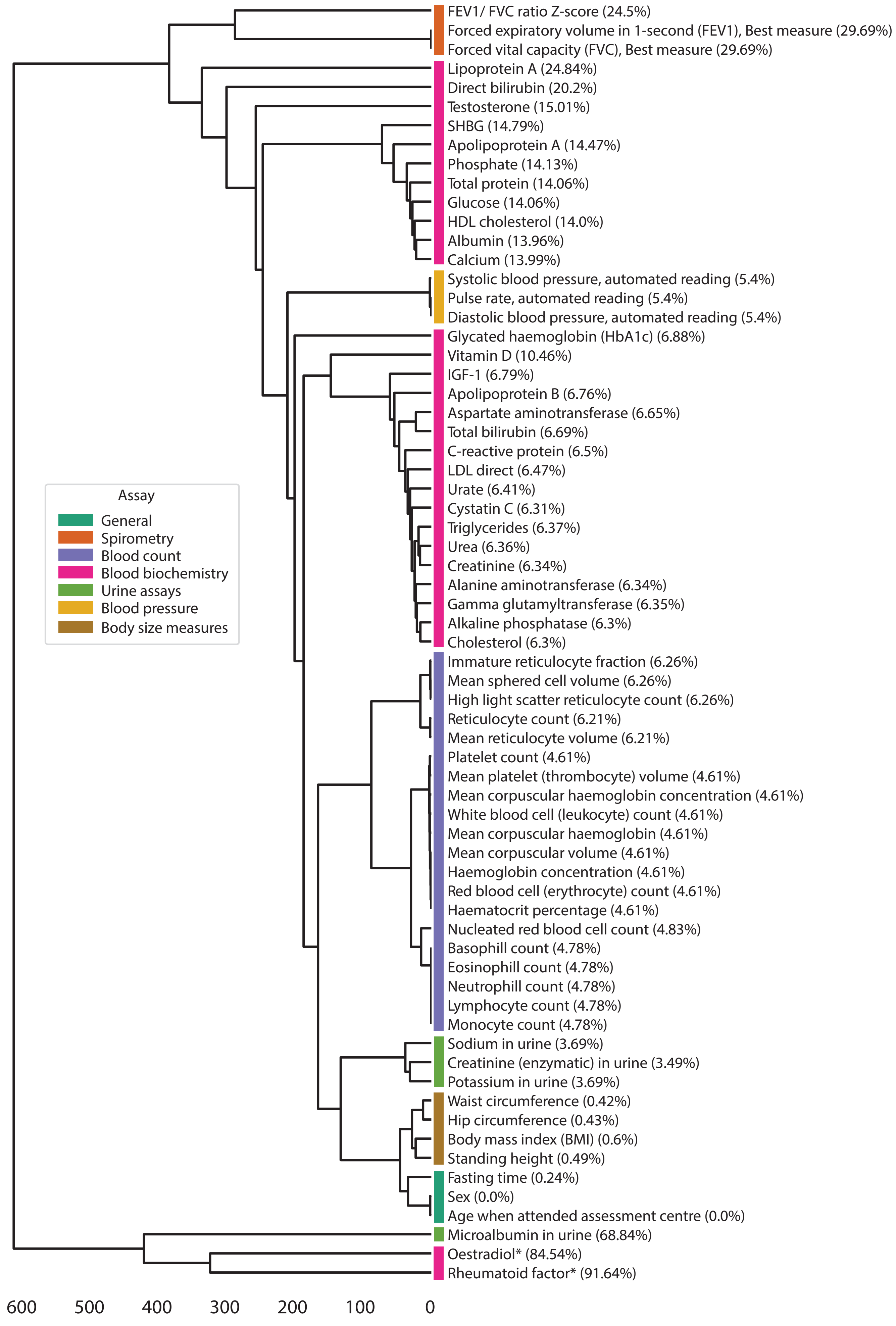

Supplementary Fig. 27 Dendrogram clustering the features based on their missingness pattern with amount of missingness indicated in brackets. * indicates features with more than 80% missing values. LDL: low density lipoprotein; IGF: insulin-like growth factor; SHBG: sex hornome binding globulin.

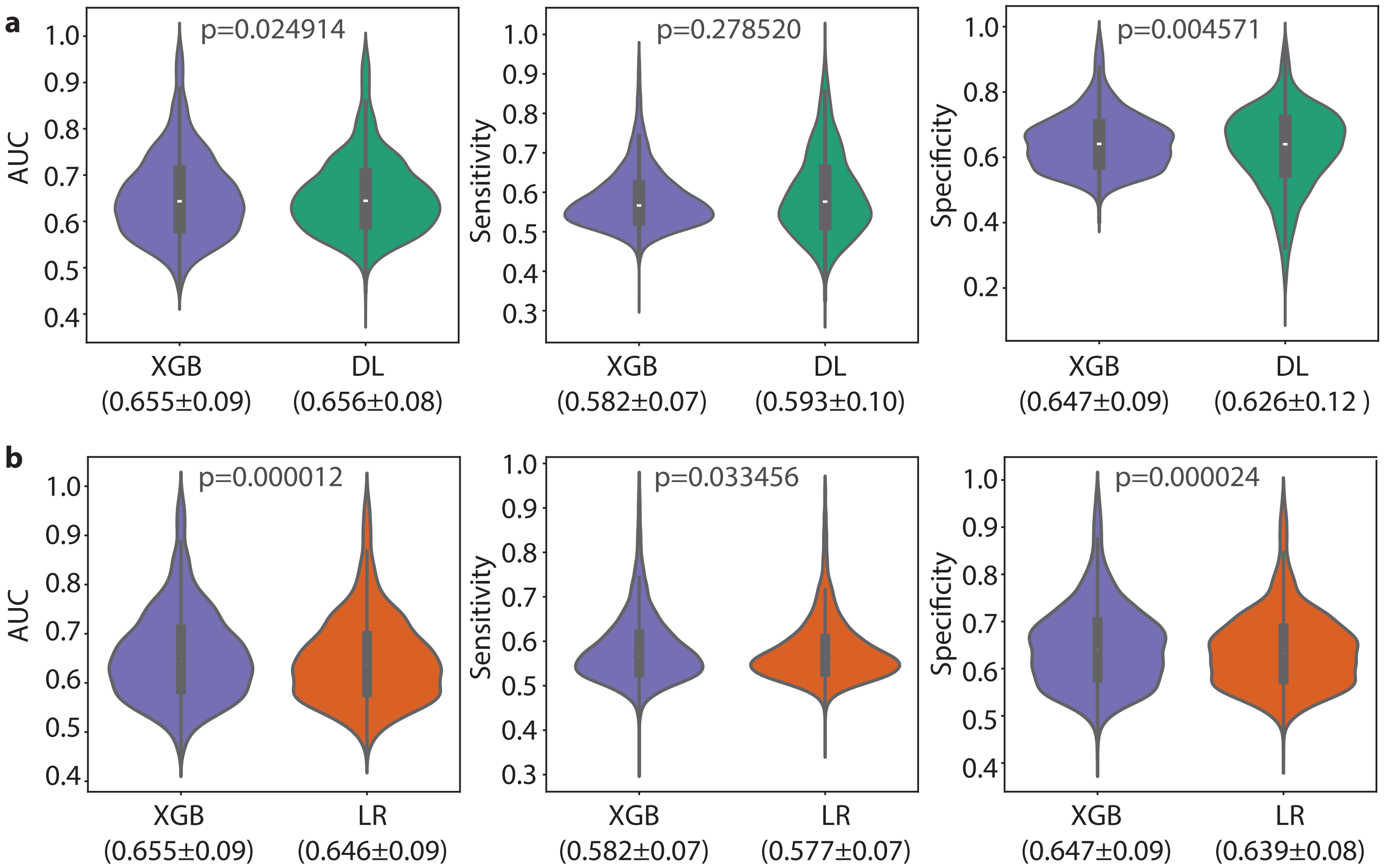

Supplementary Fig. 28 Comparison of MILTON's performance when using XGBoost vs other algorithms across ~4,250 ICD10 codes when using 67 traits (European ancestry only). Violinplots comparing AUC, sensitivity and specificity acheived for each ICD10 code when using **a** XGBoost (XGB) vs deep learning (DL) models (n=4,259), and **b** XGBoost (XGB) vs logistic regression (LR) models (n=4,278) for training MILTON. Mann Whitney two-sided p-values are shown on the top. Mean ± std of performance for each classification model is indicated in brackets on x-axis labels. Each box-plot shows median as centre line, 25th percentile as lower box limit, 75th percentile as upper box-limit, whiskers extend to 25th percentile – 1.5 * interquartile range at the bottom and 75th percentile + 1.5*interquartile range at the top, points denote outliers. Blue: XGB, green: DL, orange: LR.

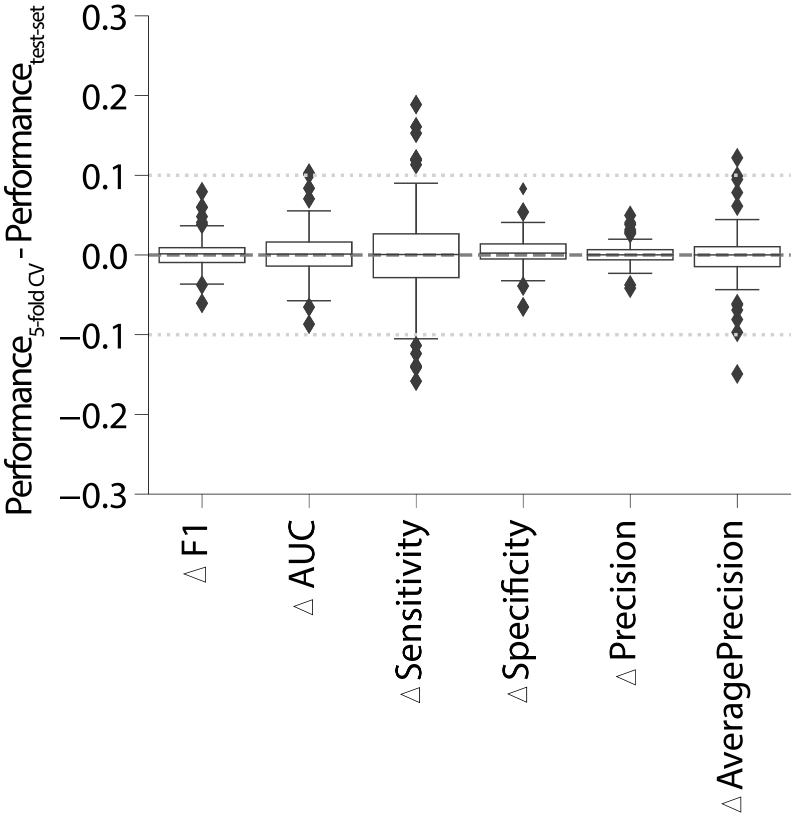

Supplementary Fig. 29 Boxplots comparing differences in various performance metrics obtained when using 5-fold cross validation (CV) vs when MILTON is only trained on 85% of data and evaluated on remaining 15% of data (held-out test set) across 100 ICD10-codes. Each box-plot shows median as centre line, 25th percentile as lower box limit, 75th percentile as upper box-limit, whiskers extend to 25th percentile – 1.5 * interquartile range at the bottom and 75th percentile + 1.5*interquartile range at the top, points denote outliers.

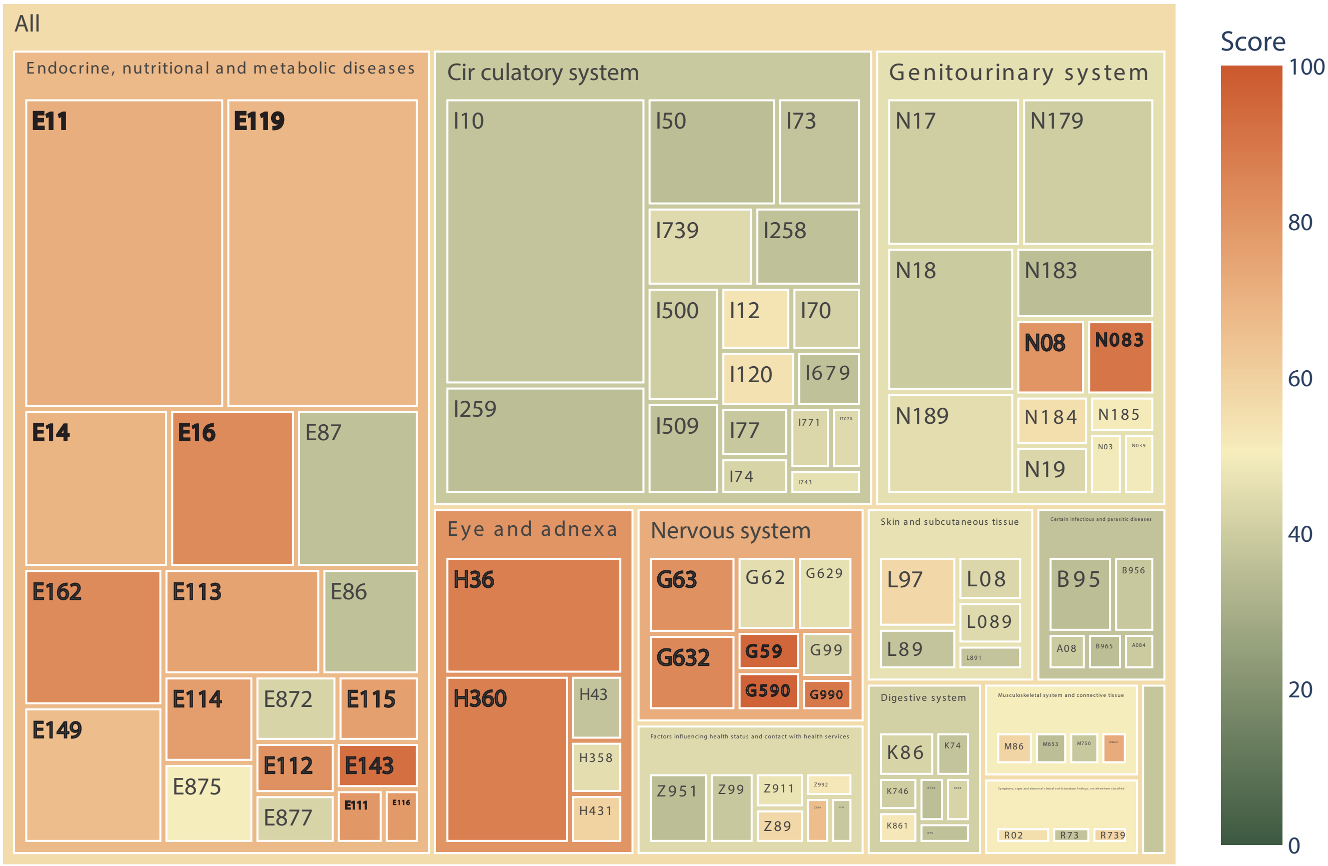

Supplementary Fig. 30 Treemap showing comorbidities in 425 patients diagnosed with E10 in UKB. The ICD10 codes with higher log odds ratio (score) compared to other ICD10 codes within each chapter are shown in dark red color. ICD10 codes that also cluster together (with E10) based on similarities in their biomarker profiles (Figure 4e) are highlighted in bold font.
