# Supplementary material for: Disease prediction with multi-omics and biomarkers empowers case–control genetic discoveries in the UK Biobank
Source: Nat Genet. 2024 Sep 11;56(9):1821–31. doi: 10.1038/s41588-024-01898-1 (PMC11390475; doi:10.1038/s41588-024-01898-1)
Supplement: Supplementary file 2 — Reporting Summary [file 41588_2024_1898_MOESM2_ESM.pdf]

Corresponding author(s): Slavé Petrovski & Dimitrios Vitsios

Last updated by author(s): 31.07.2024

Reporting Summary

Nature Portfolio wishes to improve the reproducibility of the work that we publish. This form provides structure for consistency and transparency in reporting. For further information on Nature Portfolio policies, see our Editorial Policies and the Editorial Policy Checklist.

Statistics

For all statistical analyses, confirm that the following items are present in the figure legend, table legend, main text, or Methods section.

n/a	Confirmed	
☐	☒	The exact sample size (*n*) for each experimental group/condition, given as a discrete number and unit of measurement
☐	☒	A statement on whether measurements were taken from distinct samples or whether the same sample was measured repeatedly
☐	☒	The statistical test(s) used AND whether they are one- or two-sided<br>*Only common tests should be described solely by name; describe more complex techniques in the Methods section.*
☐	☒	A description of all covariates tested
☐	☒	A description of any assumptions or corrections, such as tests of normality and adjustment for multiple comparisons
☐	☒	A full description of the statistical parameters including central tendency (e.g. means) or other basic estimates (e.g. regression coefficient) AND variation (e.g. standard deviation) or associated estimates of uncertainty (e.g. confidence intervals)
☐	☒	For null hypothesis testing, the test statistic (e.g. *F*, *t*, *r*) with confidence intervals, effect sizes, degrees of freedom and *P* value noted<br>*Give P values as exact values whenever suitable.*
☒	☐	For Bayesian analysis, information on the choice of priors and Markov chain Monte Carlo settings
☒	☐	For hierarchical and complex designs, identification of the appropriate level for tests and full reporting of outcomes
☐	☒	Estimates of effect sizes (e.g. Cohen's *d*, Pearson's *r*), indicating how they were calculated

Our web collection on statistics for biologists contains articles on many of the points above.

Software and code

Policy information about availability of computer code

Data collection	MILTON accesses UKB individual level data, therefore, users first need to apply to UK Biobank to request for access to the UKB input files. Detailed documentation is included in our MILTON public code repository to convert those files into an appropriate format, ready to be used by the MILTON package. Mock data files with the expected format are already provided in our repository for reference and testing. All generated results are available in our public results portal: http://milton.public.cgr.astrazeneca.com
Data analysis	The MILTON method is publicly available at https://zenodo.org/records/13134144 and https://github.com/astrazeneca-cgr-publications/milton-release, under the Mozilla Public License 2.0.<br><br>For data analysis and visualization, the following python packages were used: python (v3.10.13), pandas (v2.1.4), numpy (v1.22.4), matplotlib (v3.8.0), seaborn (v0.12.2), statannotations (v0.5.0), upSetPlot (v0.8.0), missingno (v0.5.1), scipy (v1.11.4). For GWAS analysis, REGENIE v3.1 was used and for quantitative PheWAS PEACOK v2.0.0 was used.

For manuscripts utilizing custom algorithms or software that are central to the research but not yet described in published literature, software must be made available to editors and reviewers. We strongly encourage code deposition in a community repository (e.g. GitHub). See the Nature Portfolio guidelines for submitting code & software for further information.

Data

Policy information about availability of data

All manuscripts must include a data availability statement. This statement should provide the following information, where applicable:

- Accession codes, unique identifiers, or web links for publicly available datasets
- A description of any restrictions on data availability
- For clinical datasets or third party data, please ensure that the statement adheres to our policy

All the biomarker information, diagnosis information along with relevant dates and whole genome sequencing data can be obtained from the UKB (http://www.ukbiobank.ac.uk/register-apply). The list of 67 quantitative traits along with their UKB field ids is given in Supplementary Table 10 and can be found on the UKB showcase portal (https://biobank.ndph.ox.ac.uk/showcase/search.cgi). UKB plasma proteomics data can also be found here: https://biobank.ndph.ox.ac.uk/showcase/label.cgi?id=1838. Data for this study were obtained under Resource Application Number 26041.

FinnGen GWAS summary statistics results can be downloaded from here: https://www.finngen.fi/en/access_results.

Baseline quantitative PheWAS results can be accessed through AZ PheWAS portal: https://www.azphewas.com.

Ensembl Human GRCh37: https://grch37.ensembl.org/Homo_sapiens/Info/Index.

All results produced in this study are available in Supplementary Tables and can be visualized on the MILTON web-portal (http://milton.public.cgr.astrazeneca.com). PheWAS/ExWAS (allelic model) results for each gene/variant as well as feature importance scores for each ICD10-code can also be downloaded by clicking on the downward arrow on their corresponding web-pagefrom the MILTON public portal. To aid with visualization, PheWAS/ExWAS results are shown for 67 biomarkers while feature importance scores are shown for 67 biomarkers with or without UKB protein expression data. All results on the portal are for European ancestry only, thatwhich comprised the majority of results (see Supplementary Tables for all ancestries). Source data corresponding to all main figures has also been provided.

Research involving human participants, their data, or biological material

Policy information about studies with human participants or human data. See also policy information about sex, gender (identity/presentation), and sexual orientation and race, ethnicity and racism.

Reporting on sex and gender	Related policy is specified in the UK Biobank project (http://www.ukbiobank.ac.uk)
Reporting on race, ethnicity, or other socially relevant groupings	Related policy is specified in the UK Biobank project (http://www.ukbiobank.ac.uk)
Population characteristics	Related policy is specified in the UK Biobank project (http://www.ukbiobank.ac.uk).<br><br>UKB cohort: The UKB comprises of data from 502,226 participants aged 37-73 years at the time of recruitment with median age being 58 years. Of these, 54.4% are females. The data collected from these participants includes, but not limited to, up-to-date diagnosis information, body size measures, blood count measures, blood biochemistry measures, genomics data as well as proteomics data (for 10% of participants). All participants provided informed consent and participation was voluntary.<br><br>FinnGen cohort: The FinnGen comprises of data from 412,181 individuals (55.9% females) with median age of 63 years. All participants provided informed consent and participation was voluntary. We did not apply for access to patient-level data and only used FinnGen GWAS summary statistics to validate our findings.
Recruitment	Related policy is specified in the UK Biobank project (http://www.ukbiobank.ac.uk)
Ethics oversight	Related policy is specified in the UK Biobank project (http://www.ukbiobank.ac.uk)

Note that full information on the approval of the study protocol must also be provided in the manuscript.

Field-specific reporting

Please select the one below that is the best fit for your research. If you are not sure, read the appropriate sections before making your selection.

☒ Life sciences ☐ Behavioural & social sciences ☐ Ecological, evolutionary & environmental sciences

For a reference copy of the document with all sections, see nature.com/documents/nr-reporting-summary-flat.pdf

Life sciences study design

All studies must disclose on these points even when the disclosure is negative.

Sample size	N=484,230 whole genome sequencing samples from UK Biobank, derived after QC checks and filtering for relatedness.

Data exclusions	We applied our method to high quality, predominantly unrelated gexome sequencing samples from 5 different ancestries with linked health record data in UKB.
Replication	We developed MILTON as an ensemble of ensemble classifiers, looking at multiple random sub-samples of the entire cohort during training, applying k-fold cross-validation, and repeating the process for 10 stochastic iterations, to increase robustness of the prediction results.
Randomization	Models are trained on all cases during each of the 10 iterations. However in each iteration, controls are randomly selected and matched to cases by size (nine times or nineteen times the number of cases), age and sex. The whole training process is repeated for 10 stochastic iterations to allow inclusion of different and diverse parts of the entire cohort during learning.
Blinding	Data collection and analysis were not performed blind to the conditions of the experiments.

Reporting for specific materials, systems and methods

We require information from authors about some types of materials, experimental systems and methods used in many studies. Here, indicate whether each material, system or method listed is relevant to your study. If you are not sure if a list item applies to your research, read the appropriate section before selecting a response.

Materials & experimental systems

n/a	Involved in the study
☒	☐ Antibodies
☒	☐ Eukaryotic cell lines
☒	☐ Palaeontology and archaeology
☒	☐ Animals and other organisms
☒	☐ Clinical data
☒	☐ Dual use research of concern
☒	☐ Plants

Methods

n/a	Involved in the study
☒	☐ ChIP-seq
☒	☐ Flow cytometry
☒	☐ MRI-based neuroimaging

Plants

Seed stocks	*Report on the source of all seed stocks or other plant material used. If applicable, state the seed stock centre and catalogue number. If plant specimens were collected from the field, describe the collection location, date and sampling procedures.*
Novel plant genotypes	*Describe the methods by which all novel plant genotypes were produced. This includes those generated by transgenic approaches, gene editing, chemical/radiation-based mutagenesis and hybridization. For transgenic lines, describe the transformation method, the number of independent lines analyzed and the generation upon which experiments were performed. For gene-edited lines, describe the editor used, the endogenous sequence targeted for editing, the targeting guide RNA sequence (if applicable) and how the editor was applied.*
Authentication	*Describe any authentication procedures for each seed stock used or novel genotype generated. Describe any experiments used to assess the effect of a mutation and, where applicable, how potential secondary effects (e.g. second site T-DNA insertions, mosiacism, off-target gene editing) were examined.*
